# Synthetic retinoid-mediated preconditioning of cancer-associated fibroblasts and macrophages improves cancer response to immune checkpoint blockade

**DOI:** 10.1038/s41416-024-02734-3

**Published:** 2024-06-07

**Authors:** Takayuki Owaki, Tadashi Iida, Yuki Miyai, Katsuhiro Kato, Tetsunari Hase, Makoto Ishii, Ryota Ando, Kunihiko Hinohara, Tomohiro Akashi, Yasuyuki Mizutani, Takuya Ishikawa, Shinji Mii, Yukihiro Shiraki, Nobutoshi Esaki, Masami Yamamoto, Tetsuya Tsukamoto, Sachiyo Nomura, Takashi Murakami, Masahide Takahashi, Yuri Yuguchi, Motohiro Maeda, Tomoyasu Sano, Naoto Sassa, Yoshihisa Matsukawa, Hiroki Kawashima, Shusuke Akamatsu, Atsushi Enomoto

**Affiliations:** 1 Department of Pathology, Nagoya University Graduate School of Medicine, Nagoya, Japan; 2 Department of Urology, Nagoya University Graduate School of Medicine, Nagoya, Japan; 3 Department of Gastroenterology and Hepatology, Nagoya University Graduate School of Medicine, Nagoya, Japan; 4 Department of Cardiology, Nagoya University Graduate School of Medicine, Nagoya, Japan; 5 Department of Respiratory Medicine, Nagoya University Graduate School of Medicine, Nagoya, Japan; 6 Department of Immunology, Nagoya University Graduate School of Medicine, Nagoya, Japan; 7 Institute for Advanced Research, Nagoya University, Nagoya, Japan; 8 Division of Systems Biology, Graduate School of Medicine, Nagoya University, Nagoya, Japan; 9 Laboratory of Physiological Pathology, Nippon Veterinary and Life Science University, Tokyo, Japan; 10 Division of Analytical Pathology, Oncology Innovation Center, Fujita Health University, Toyoake, Japan; 11 Department of Gastrointestinal Surgery, Graduate School of Medicine, The University of Tokyo, Tokyo, Japan; 12 Department of Clinical Pharmaceutical Sciences, School of Pharmacy and Pharmaceutical Sciences, Hoshi University, Tokyo, Japan; 13 Department of Microbiology, Saitama Medical University, Saitama, Japan; 14 Department of Pathology, Fujita Health University, Toyoake, Japan; 15 International Center for Cell and Gene Therapy, Fujita Health University, Toyoake, Japan; 16 Department of Urology, Chukyo Hospital, Nagoya, Japan; 17 Department of Urology, Aichi Medical University, Nagakute, Japan; 18 Center for One Medicine Innovative Translational Research, Gifu University Institute for Advanced Study, Gifu, Japan

**Keywords:** Translational research, Cancer microenvironment

## Abstract

**Background:**

The proliferation of cancer-associated fibroblasts (CAFs) hampers drug delivery and anti-tumor immunity, inducing tumor resistance to immune checkpoint blockade (ICB) therapy. However, it has remained a challenge to develop therapeutics that specifically target or modulate CAFs.

**Methods:**

We investigated the involvement of Meflin^+^ cancer-restraining CAFs (rCAFs) in ICB efficacy in patients with clear cell renal cell carcinoma (ccRCC) and urothelial carcinoma (UC). We examined the effects of Am80 (a synthetic retinoid) administration on CAF phenotype, the tumor immune microenvironment, and ICB efficacy in cancer mouse models.

**Results:**

High infiltration of Meflin^+^ CAFs correlated with ICB efficacy in patients with ccRCC and UC. Meflin^+^ CAF induction by Am80 administration improved ICB efficacy in the mouse models of cancer. Am80 exerted this effect when administered prior to, but not concomitant with, ICB therapy in wild-type but not Meflin-deficient mice. Am80-mediated induction of Meflin^+^ CAFs was associated with increases in antibody delivery and M1-like tumor-associated macrophage (TAM) infiltration. Finally, we showed the role of Chemerin produced from CAFs after Am80 administration in the induction of M1-like TAMs.

**Conclusion:**

Our data suggested that Am80 administration prior to ICB therapy increases the number of Meflin^+^ rCAFs and ICB efficacy by inducing changes in TAM phenotype.

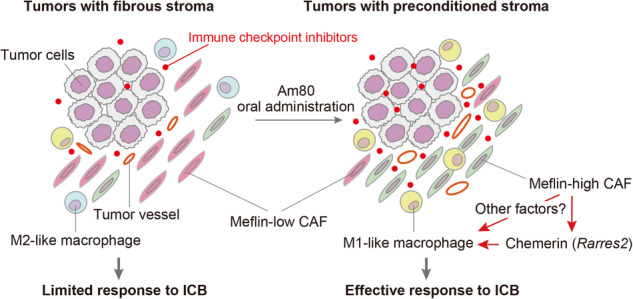

## Introduction

Cancer-associated fibroblasts (CAFs) are one of the major cell subsets in the tumor microenvironment [1–11]. Their proliferation is observed in almost all invasive cancers but is most prominent in aggressive cancers with marked desmoplastic stromal reactions, such as pancreatic ductal adenocarcinoma (PDAC) [4, 8, 11]. CAFs secrete a number of cytokines, chemokines, growth factors, and exosomes that promote cancer cell proliferation and motility and suppress the anti-tumor immune response [1–10]. They also produce many types of extracellular matrix (ECM) components, which induces fibrosis and increases interstitial pressure and tumor stiffness [1–11]. These changes in the physical properties of the tumor stroma increase the malignancy of tumor cells and result in poor drug delivery as they induce the collapse of tumor vessels [11].

Recent single-cell transcriptomic analyses have revealed CAF heterogeneity, the extent of which and the best clustering method vary depending on cancer types and species [1, 3–5, 8, 10]. One approach to classify CAFs is based on their cellular roles in cancer progression: cancer-promoting CAFs (pCAFs) and cancer-restraining CAFs (rCAFs) [2, 3, 9, 12, 13]. One of the issues with this classification, however, is that there are limited reports on fibroblast-specific markers that functionally define rCAFs. A functional marker of rCAFs is collagen type I alpha I (Col1α1), which is a major component of the ECM [3, 14, 15]. The genetic deletion of the *Col1a1* gene in CAFs expressing α-smooth muscle actin (α-SMA) results in the suppression of anti-tumor immunity and tumor progression in a PDAC mouse model [14, 15]. In contrast, Col1α1 homotrimer produced by cancer cells promotes cancer progression, making it challenging to determine the net effect of total Col1α1 [16].

Another rCAF marker is Meflin, a glycosylphosphatidylinositol-anchored membrane or secreted protein encoded by the immunoglobulin superfamily containing leucine-rich repeat (*Islr*) gene [12, 13, 17]. Meflin was previously identified as a marker specific for normal perivascular fibroblasts or mesenchymal stem cells (MSCs) that are present across various tissues in both humans and mice [18–20]. The function of Meflin is to suppress tissue fibrosis through augmentation of bone morphogenetic protein 7 (BMP7) signaling and inhibition of the collagen cross-linking activity of lysyl oxidase (Lox) [21–25]. Meflin inhibits the differentiation of normal fibroblasts into α-SMA-positive (α-SMA^+^) myofibroblasts or myofibroblastic CAFs (myCAFs) [17, 21]. In contrast, Meflin expression in normal fibroblasts is rapidly downregulated when they differentiate into myofibroblasts or myCAFs in response to exposure to transforming growth factor-β (TGF-β), stiff substrate, or hypoxic conditions [21, 23]. A lineage-tracing experiment in mice confirmed the state transition of Meflin^+^ normal fibroblasts into myCAFs during tumor progression [17]. An autochthonous murine model of PDAC (KPC model) exhibited accelerated tumor progression when the model mice were crossed with Meflin knockout (KO) mice [17]. The number of Meflin^+^ CAFs was correlated with better outcomes in patients with advanced stages of PDAC and colorectal cancer (CRC) [17, 25]. Taken together, it was proposed that Meflin is a functional marker of rCAFs [12, 13, 17, 20, 25].

We recently identified Am80 (trade name: tamibarotene), a synthetic retinoid that has higher selectivity for retinoic acid (RA) receptors than naturally occurring all-trans-retinoic acid (ATRA), as a compound that upregulates Meflin expression in CAFs [24]. Am80 possesses unique chemical properties, including excellent physical stability and low affinity for binding with cellular retinoic acid binding proteins (CRABPs) compared to ATRA, making it clinically useful for the treatment of patients with acute promyelocytic leukemia [26, 27]. We reported that oral administration of Am80 increased Meflin^+^ rCAFs, decreased α-SMA^+^ myCAFs, and suppressed stromal fibrosis in PDAC developed in mice [24]. This was accompanied by a decrease in tumor stiffness and increases in tumor vessel area and drug delivery [24]. The combination therapy of Am80 and conventional cytotoxic anti-cancer agents improved the outcomes of PDAC developed in wild-type (WT) mice, but not Meflin KO mice [24]. These data suggest that CAF conditioning or reprogramming by Am80 may be a promising strategy to improve the efficacy of conventional anti-cancer agents.

Interestingly, another previous study demonstrated that the number of Meflin^+^ rCAFs positively correlated with the response to immune checkpoint blockade (ICB) therapy in patients with non-small cell lung cancer (NSCLC) [28]. This was also shown in mouse models, where tumors developed in Meflin KO mice and mice that overexpress Meflin showed lower and higher sensitivity to anti-PD1 antibody treatment, respectively [28]. These data suggest the significance of the balance between pCAFs and rCAFs in ICB therapy, being consistent with recent studies that have shown the critical role of CAFs in anti-tumor immunity [5–7, 29],

In the present study, we confirmed the significance of the pCAF/rCAF balance in the response to ICB therapy in patients with advanced urological cancers. We then investigated the effect of Am80 administration on the efficacy of anti-PD-L1 antibody treatment using murine models of urothelial carcinoma (UC) and PDAC. Intriguingly, we found that the timing of Am80 administration was crucial when aiming to improve the efficacy of anti-PD-L1 antibody treatment, different from conventional cytotoxic anti-cancer agents. We also addressed the mechanism by which Meflin^+^ rCAFs shape the tumor immune microenvironment (TIME) that potentiates the efficacy of ICB therapy.

## Results

### Meflin expression in CAFs correlates with ICB efficacy in urological cancers

**Fig. 1:**
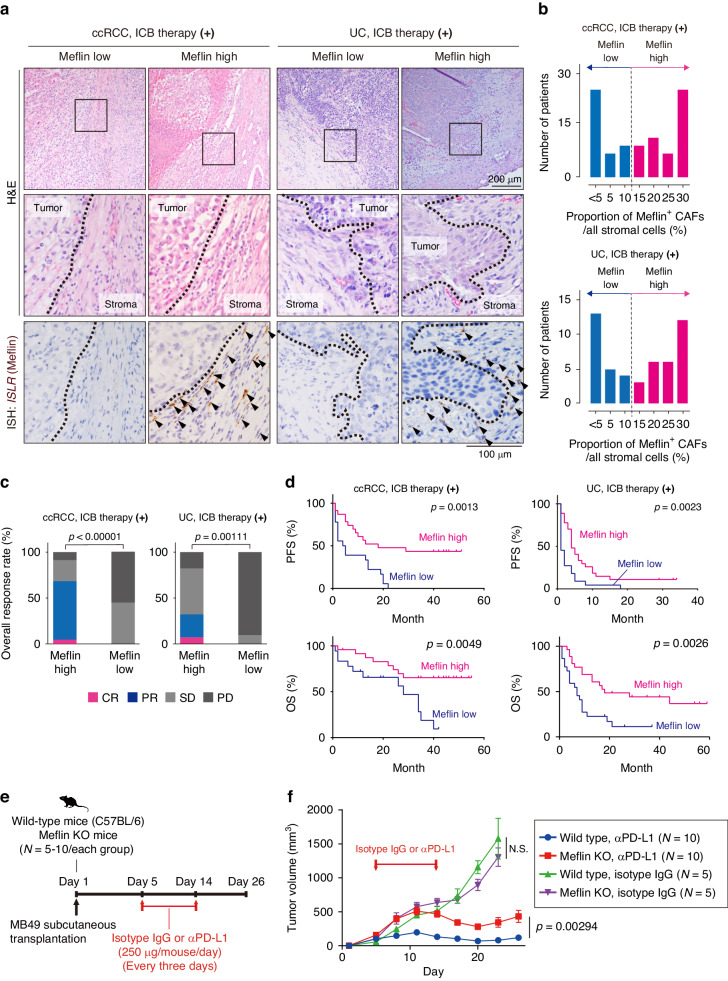
Expression of Meflin in CAFs in ccRCC and UC and its significance to ICB therapy. **a** Tissue sections obtained from human ccRCC and UC cases who underwent ICB therapy were hematoxylin and eosin stained (top panels), and stained for Meflin mRNA by ISH (lower panels). Boxed areas are magnified in the middle panels. Dotted lines indicate the boundaries between the tumor and the stroma. Arrowheads indicate Meflin^+^ CAFs. **b** Patients with ccRCC (top) or UC (bottom) treated with ICB therapy were stratified by the numbers of Meflin^+^ CAFs in the stromal tissue. **c** Objective response rate (ORR) of Meflin-high and -low ccRCC and UC cases who received ICB therapy. **d** PFS (top) and OS (bottom) of Meflin-high and -low ccRCC or UC patients treated with ICB therapy. **e**, **f** C57BL/6 wild-type and Meflin KO female mice were subcutaneously transplanted with MB49 cells (1 × 10^6^ cells/mouse) on Day 1, followed by intraperitoneal injection of anti-PD-L1 (αPD-L1) or isotype control IgG every three days from Day 5 to Day 14 (**e**). Tumor volume (**f**). The statistical methods used are a two-tailed unpaired t-test (**c**, **f**) and log-rank (Mantel–Cox) test (**d**). CAFs cancer-associated fibroblasts, ccRCC clear cell renal cell carcinoma, CR complete response, ICB immune checkpoint blockade, Ig immunoglobulin, ISH in situ hybridization, KO knockout, N.S. not significant, OS overall survival, PD progressive disease, PD-L1 programmed cell death ligand 1, PFS progression-free survival, PR partial response, SD stable disease, UC: urothelial carcinoma.

We first examined the association between the number of Meflin^+^ CAFs and the outcomes of patients with unresectable clear cell renal cell carcinoma (ccRCC) and UC, who were treated with either conventional chemotherapy (Supplementary Tables S1 and S2) or ICB therapy (Table 1 and Supplementary Table S3). An analysis of publicly available single-cell transcriptome data (GSA accession no. HRA000212, ref. [30]) showed that Meflin expression was enriched in CAFs of human UC, supporting the notion that Meflin is a specific marker of CAFs (Supplementary Fig. S1). Meflin, α-SMA (*Acta2*), and interleukin-6 (IL-6) were expressed in different CAF subsets, consistent with the findings of previous studies suggesting that Meflin^+^ CAFs are distinct from myCAFs or inflammatory CAFs (iCAFs) in PDAC, CRC, and NSCLC [12, 17, 25, 28, 31]. Meflin expression in CAFs was confirmed by in situ hybridization (ISH) on tumor tissue sections obtained from patients with ccRCC and UC. This also revealed variable proliferation and distribution of *ISLR* (Meflin)^+^ CAFs between the patients (Fig. 1a). To quantify Meflin^+^ CAFs, we assigned all stromal cells with oval- to spindle-shaped nuclei as CAFs and counted the number of Meflin^+^ cells, following the method described previously [28]. Subsequently, patients were stratified into Meflin-high and low groups using the threshold of Meflin-high as ≥15% CAFs expressing Meflin (Fig. 1b and Supplementary Fig. S2A).

**Table 1:** Hazard ratios and *P* values for multivariate Cox proportional hazard regression model analysis in patients with ccRCC and UC treated with ICB therapy.

Variable	Hazard ratio (95% CI) for OS	*P* value for OS	Hazard ratio (95% CI) for PFS	*P* value for PFS
**(a) Hazard ratios and** ***P*** **values for multivariate Cox proportional hazard regression modelanalysis in patients with ccRCC treated with ICB therapy**				
Age at surgery		0.218		0.136
≤65	Reference		Reference	
65<	0.4949 (0.16170–1.514)		0.5111 (0.2118–1.2340)	
Sex		0.671		0.108
Male	Reference		Reference	
Female	1.3450 (0.34290–5.272)		2.5000 (0.8175–7.6440)	
TNM stage		0.048		0.892
≤3	Reference		Reference	
4	3.0830 (1.01200–9.395)		0.9487 (0.4445–2.0250)	
ECOG-PS		0.049		0.061
0 or 1 (Good)	Reference		Reference	
2≤ (Poor)	8.6550 (1.00900–74.230)		5.8390 (0.9193–37.0800)	
Treatment line		0.006		0.010
1^st^	Reference		Reference	
2^nd^ or 3^rd^	10.2400 (1.93600–54.160)		3.0590 (1.3050–7.1710)	
Meflin Expression		0.008		0.023
Low	Reference		Reference	
High	0.2566 (0.09405–0.700)		0.4064 (0.1862–0.8869)	
**(b) Hazard ratios and** ***P*** **values for multivariate Cox proportional hazard regression modelanalysis in patients with UC treated with ICB therapy**				
Age at surgery		0.735		0.482
≤65	Reference		Reference	
65<	1.1650 (0.48170–2.8170)		1.3120 (0.6157–2.7950)	
Sex		0.021		0.147
Male	Reference		Reference	
Female	0.2711 (0.08959–0.8200)		0.5116 (0.2070–1.2650)	
Primary site		0.016		0.216
Bladder	Reference		Reference	
Upper urinary tract	2.8060 (1.21200–6.4930)		1.5500 (0.7747–3.0990)	
TNM stage		0.628		0.368
0 or 1	Reference		Reference	
2≤	1.3430 (0.40780–4.4230)		1.5410 (0.6007–3.9560)	
Neoadjuvant chemotherapy		0.783		0.370
No	Reference		Reference	
Yes	0.8690 (0.32050–2.3560)		0.6679 (0.2764–1.6140)	
Brinkman index		0.129		0.180
<400	Reference		Reference	
400≤	0.4846 (0.19040–1.2330)		0.5857 (0.2678–1.2810)	
ECOG-PS		<0.001		0.728
0 or 1 (Good)	Reference		Reference	
2≤ (Poor)	5.9790 (2.10900–16.9500)		1.1720 (0.4795–2.8660)	
Meflin Expression		0.008		0.024
Low	Reference		Reference	
High	0.3054 (0.12650–0.7371)		0.4183 (0.1965–0.8905)

The analysis of the overall survival of patients with ccRCC and UC treated with conventional chemotherapy revealed that the Meflin-high groups tended to have worse outcomes than the Meflin-low groups (Supplementary Fig. S2B and Supplementary Table S1). However, the analysis of patients with ccRCC and UC treated with ICB therapy showed that the Meflin-high groups showed a better objective response rate (ORR), progression-free survival (PFS), and overall survival (OS) than the Meflin-low groups (Fig. 1c, d and Table 1). The data were consistent with our previous findings that high numbers of Meflin^+^ CAFs are associated with extensive desmoplastic stromal reactions, advanced stage, and poor prognosis, but better therapeutic response to ICB therapy in NSCLC [28].

To confirm the significance of Meflin expression in CAFs in response to ICB therapy, we subcutaneously transplanted C57BL/6 WT and Meflin KO female mice with MB49, a syngeneic urothelial carcinoma cell line [32], followed by intraperitoneal (i.p.) administration of anti-PD-L1 antibody or isotype control IgG (Fig. 1e). The growth rate of MB49 tumors was comparable between WT and Meflin KO mice. However, the effect of anti-PD-L1 antibody treatment was significantly abrogated in Meflin KO mice (Fig. 1f). These data suggested a pivotal role of Meflin expression in CAFs in the tumor response to ICB therapy.

### Pharmacological induction of Meflin expression in CAFs improves ICB efficacy

We previously identified a synthetic retinoid Am80 as a reagent that upregulates Meflin expression in CAFs and converts Meflin^low^α-SMA^high^ rCAFs into Meflin^high^α-SMA^low^ pCAFs [24]. In syngeneic murine tumor models generated by subcutaneously or orthotopically implanting the murine PDAC cell line mT5, oral administration of Am80 increased the number of Meflin^+^ CAFs, accompanied by a decrease in tumor stiffness and increases in tumor vessel area and intratumoral delivery of the chemotherapeutic agent gemcitabine [24]. This was observed only in PDAC developed in WT but not in Meflin KO mice [24]. Therefore, our present study first focused on the effects of Am80 administration on ICB efficacy using the same PDAC model.

**Fig. 2:**
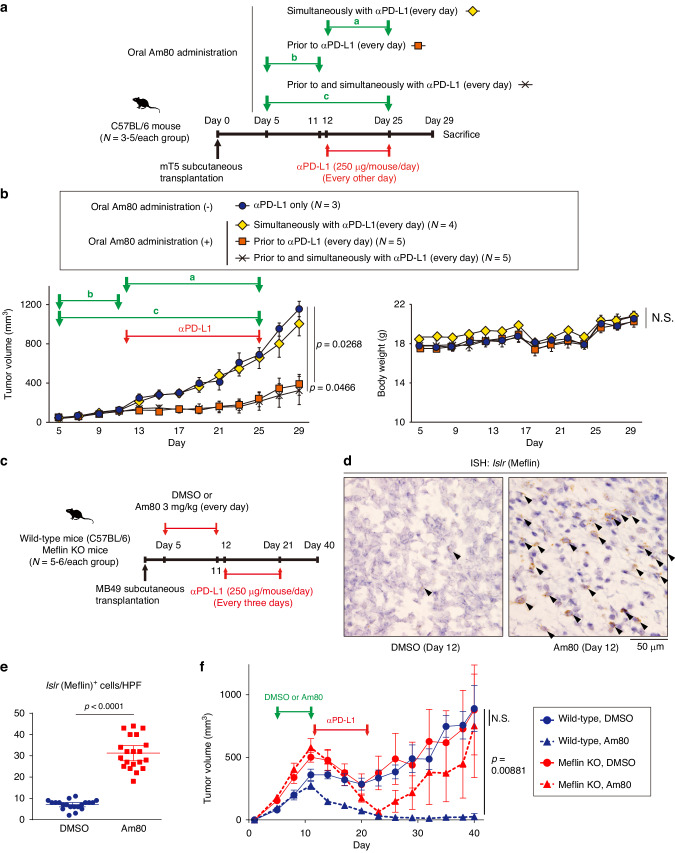
A priori oral Am80 administration enhances the efficacy of anti-PD-L1 antibody treatment in PDAC and UC mouse models. **a** C57BL/6 wild-type female mice were subcutaneously transplanted with mT5 cells (1 × 10^6^ cells/mouse), followed by oral Am80 administration at the indicated periods and intraperitoneal injection of anti-PD-L1 antibodies. a, b, and c indicate the periods for oral Am80 administration. **b** Time courses of the volume of tumors developed in mice treated by the indicated regimens (left) and their body weights (right). **c** C57BL/6 wild-type or Meflin KO female mice were subcutaneously transplanted with MB49 cells (1 × 10^6^ cells/mouse), followed by oral administration of DMSO or Am80 prior to intraperitoneal injection of anti-PD-L1 antibodies. **d**, **e** Tissue sections obtained from the MB49 tumors on Day 12 were stained for the *Islr* gene by ISH (**d**), followed by quantification of *Islr*^+^ cells (**e**). Arrowheads in (**d**) indicate *Islr*^+^ cells. **f** Time courses of the volumes of tumors of the indicated groups. Differences between groups were analyzed using 1-way ANOVA with the Tukey test (**b**), and Student t-test (**e**, **f**). HPF high-power field (×40 objective lens), ICB immune checkpoint blockade, ISH in situ hybridization, KO knockout, NS not significant, PDAC pancreatic ductal adenocarcinoma, PD-L1 programmed cell death ligand 1, UC urothelial carcinoma.

We found that mT5 syngeneic subcutaneous tumors were resistant to i.p. injection of anti-PD-L1 antibodies, consistent with previous preclinical and clinical studies in PDAC (data not shown) [33]. Simultaneous oral Am80 administration with anti-PD-L1 antibodies also did not exert tumor-suppressive effects (Fig. 2a, b). However, it was surprising but interesting to find that Am80 administration prior to the injection of anti-PD-L1 antibodies significantly boosted its effects (Fig. 2a, b). Continued Am80 administration prior to and simultaneously with anti-PD-L1 antibody treatment was also effective but did not show any additive effect compared to only prior administration of Am80 (Fig. 2a, b). Am80 monotherapy had no anti-tumor effects, and neither of the regimens had detrimental effects on body weight (Fig. 2b).

We next examined the effects of Am80 on anti-PD-L1 therapy in the MB49 UC model. First, we confirmed that the proliferation of cultured MB49 cells was not affected by Am80 at a concentration range of 1 – 10 µM, as also previously observed with mT5 cells (Supplementary Fig. S3A, B) [24]. As with mT5 tumors, Am80 administration prior to anti-PD-L1 therapy was shown to delay tumor growth better than the simultaneous administration of Am80 with anti-PD-L1 antibodies in the MB49 subcutaneous tumor model (Supplementary Fig. S4A, B, Groups a and b). The growth of MB49 tumors temporarily responded to the injection of anti-PD-L1 antibodies, but an accelerated regrowth was observed following the discontinuation of anti-PD-L1 therapy (Supplementary Fig. S4A, B, Group a). Interestingly, the injection of anti-PD-L1 antibodies at an early stage of tumor growth (Day 8) showed high therapeutic activity regardless of the timing of Am80 administration, indicating that MB49 tumors become increasingly resistant to anti-PD-L1 therapy over time (Supplementary Fig. S4A, B, Groups c and d). The data also suggested that there may be an optimal window for considering the delivery of Am80 and anti-PD-L1 antibodies in the MB49 model, which was taken into account in the following analyses.

We next found that oral Am80 administration prior to i.p. injection of anti-PD-L1 antibodies significantly increased the number of Meflin^+^ CAFs in the developed tumors (Fig. 2c–e). Am80 administration at 3.0 mg/kg daily for 7 days prior to anti-PD-L1 therapy that was initiated on Day 12 significantly improved the efficacy of anti-PD-L1 therapy and induced durable responses, resulting in complete regression in most mice (Fig. 2f). We determined that the optimal dose of Am80 was 1.0–3.0 mg/kg daily for 7 days when Am80 was administered prior to anti-PD-L1 therapy (Supplementary Fig. S4C, D). Notably, this effect of Am80 was not observed in MB49 tumors developed in Meflin KO mice; their growth was temporarily affected by anti-PD-L1 therapy, but tumors regrew shortly after the discontinuation of anti-PD-L1 therapy (Fig. 2f). The effects of Am80 administration prior to ICB therapy were also observed in the syngeneic lung and gastric cancer mouse models (Supplementary Fig. S5A–D). These data suggest that preconditioning of CAFs by Am80 prior to, but not concomitant with, ICB therapy has the potential to augment its therapeutic efficacy in various types of murine cancer models.

Considering that our previous study showed that Am80-mediated increases in Meflin expression in CAFs resulted in increases in tumor blood vessel area and intratumoral drug delivery of gemcitabine [24], we examined intratumoral delivery of i.p.-injected anti-PD-L1 rat antibodies by immunofluorescent (IF) staining on tumor tissue sections obtained from the MB49 model (Supplementary Fig. S6A). This revealed that the area stained by anti-rat IgG antibody was significantly increased by Am80 administration in tumors developed in WT mice but not Meflin KO mice (Supplementary Fig. S6B, C). These data suggest that Am80 enhances antibody delivery to tumors, which was partly mediated by an increase in Meflin expression in CAFs.

### Am80 administration induces polarization of macrophages to the M1-like phenotype in murine models of PDAC and UC

**Fig. 3:**
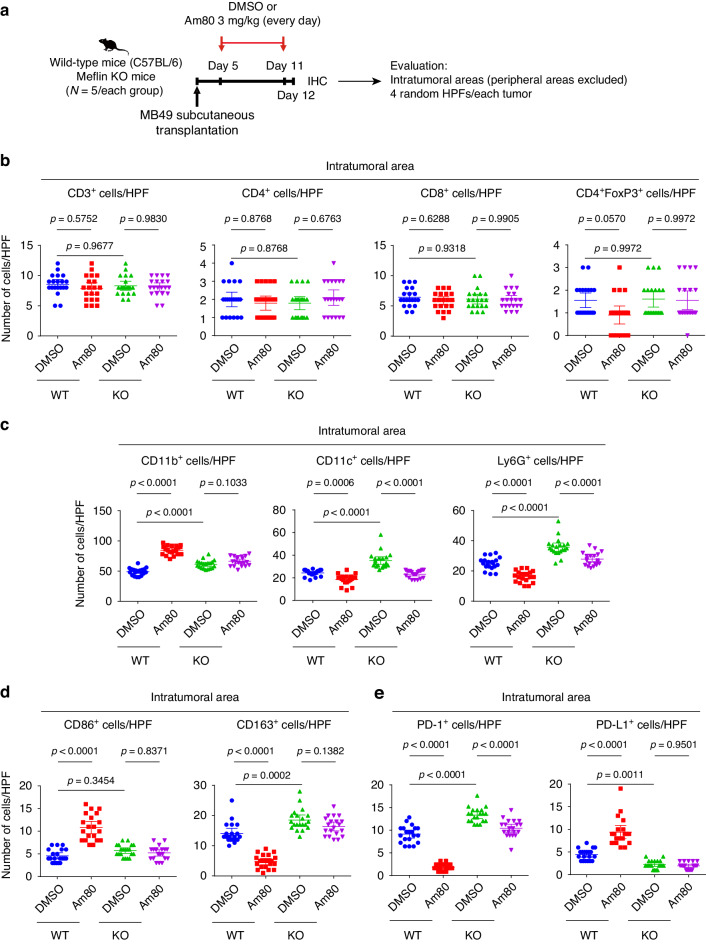
Effects of Am80 administration on the TIME in the MB49 model. **a** C57BL/6 wild-type or Meflin KO female mice were subcutaneously transplanted with MB49 cells (1 × 10^6^ cells/mouse), followed by oral administration of DMSO or Am80 and IHC for several immune cell markers. Four random HPFs from the intratumoral area for each tissue section were evaluated for quantification. **b**–**e** Cells positive for the indicated immune cell markers in each HPF were counted, followed by quantification. Twelve HPFs were evaluated for each group. The statistical methods used were 1-way ANOVA with the Tukey test (**b**–**e**). HPF high-power field (×40 objective lens), IHC immunohistochemistry, KO knockout, TIME tumor immune microenvironment.

We next performed immunohistochemistry (IHC) to examine alterations in the TIME induced by Am80 administration prior to ICB therapy (Fig. 3a). Evaluation of the intratumoral area, but not the peritumor area, which is often difficult to be discriminated with the surrounding dermis and fascia of the panniculus carnosus muscle, showed that the infiltration of myeloid cells, which were positive for CD11b, CD11c, or Ly6G, but not CD3^+^, CD4^+^ or CD8^+^ T cells, were significantly and differentially altered by Am80 administration (Fig. 3b, c and Supplementary Figs. S7, S8). The number of regulatory T cells was not altered in response to Am80 in MB49 tumors developed in WT mice (Fig. 3b). We then assessed CD11b^+^ myeloid cells, which comprise tumor-associated macrophages (TAMs), dendritic cells, and myeloid-derived suppressor cells, since their absolute numbers were relatively high, as infiltration was upregulated by Am80 in MB49 tumors developed in WT mice but not Meflin KO mice. Further IHC analyses revealed an increase in CD86^+^ M1-like TAMs but a decrease in CD163^+^ M2-like TAMs in Am80-administered MB49 tumors developed in WT mice but not in Meflin KO mice (Fig. 3d and Supplementary Fig. S8). We also found that the number of cells expressing PD-1, a co-inhibitory checkpoint molecule and a marker of T cell exhaustion [34], was significantly decreased, whereas that of PD-L1^+^ cells, which were considered to be non-tumor cells based on morphology, was increased in response to Am80 in tumors developed in WT mice but not Meflin KO mice (Fig. 3e and Supplementary Fig. S8). These data suggest that the Am80-mediated conversion of CAFs significantly affects the TIME.

The Am80-induced alteration of the TIME was also examined in the mT5 PDAC mouse model using single-cell transcriptomic analysis (Supplementary Fig. S9A). While no global changes in cell clusters were observed, gene expression analysis of T cell and TAM clusters showed that several key immunoregulatory marker genes were differentially expressed between the control and Am80 groups (Supplementary Fig. S9B, C). Consistent with the data obtained with the MB49 model, *Nos2* (iNOS)^+^ M1-like and *Mrc1* (CD206)^+^ M2-like TAMs were increased and decreased in the Am80 group compared to the control groups, respectively. This was further confirmed by IHC for macrophage markers, which showed that the infiltrated TAMs were skewed toward the M1-like phenotype in the Am80 group (Supplementary Fig. S10A, B). Unlike the MB49 model, Am80 administration had variable effects on the infiltration of T cells, including regulatory T cells, in the mT5 model, suggesting differences in Am80 effects on the TIME among different tumor types (Supplementary Fig. S9B).

Our data showed the significance of Meflin expression in CAFs in mediating the effects of Am80 in enhancing the efficacy of ICB therapy. However, it is plausible that lipophilic Am80 affects not only CAFs but various types of cells, including tumor cells. ISH on tissue sections obtained from mT5 tumors after administration of Am80 showed that the expression of *Cyp26b1*, the gene encoding an RA-metabolizing enzyme, the expression of which is activated by RA [35], was mainly upregulated in spindle-shaped stromal cells, but not in mT5 tumor cells in response to Am80 administration, supporting the notion that Am80 does not directly target tumor cells in these murine cancer models (Supplementary Fig. S10C).

### Correlation of the number of Meflin^+^ CAFs with macrophage phenotype in human urological cancers

**Fig. 4:**
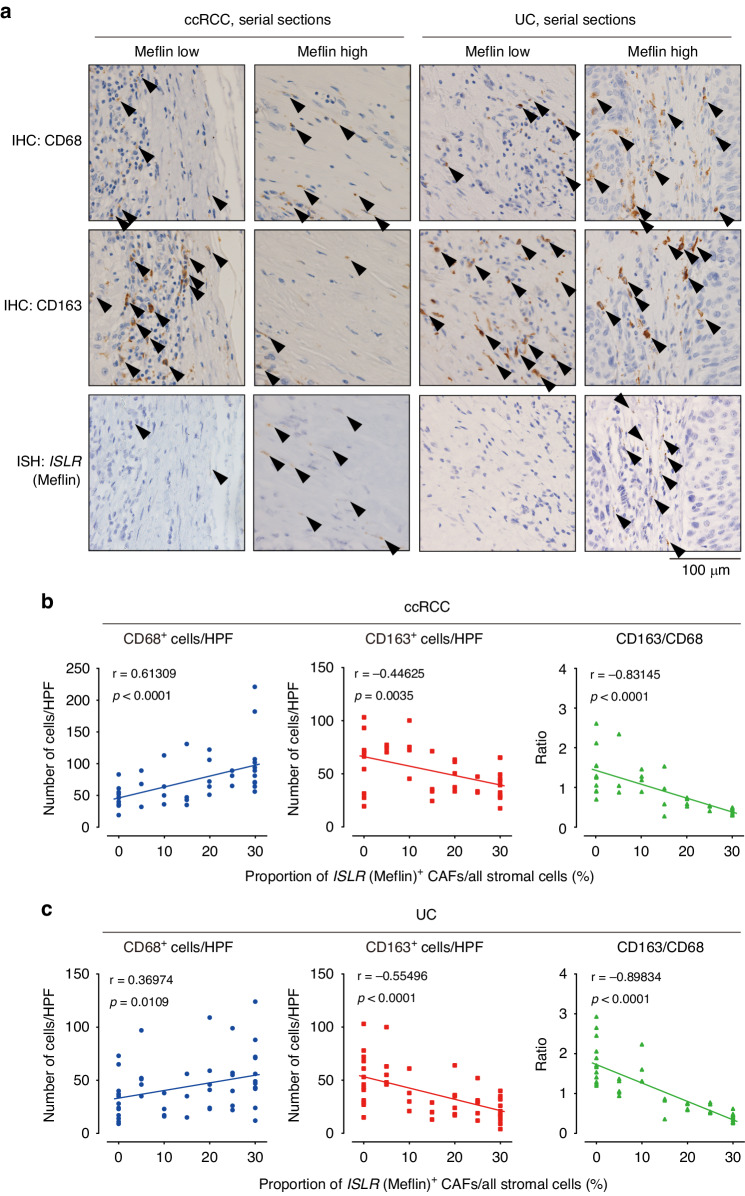
Correlation between the number of Meflin^+^ CAFs and CD68^+^ macrophages in human ccRCC and UC. **a** Serial sections prepared from surgically resected human ccRCC and UC tumors were stained for CD68 and CD163 by IHC and *ISLR* by ISH. Representative images of Meflin-low and -high cases are shown. Arrowheads denote cells positive for the indicated markers. **b**, **c** Number of cells positive for CD68 and CD163 in human ccRCC (**b**) and UC (**c**) tumor samples were counted and plotted against the proportion of Meflin^+^ CAFs in the stroma. The right panels show the correlation between the CD163/CD68 ratio and the number of Meflin^+^ CAFs. Quantitative interrelationships were determined using the Spearman’s rank-order correlation coefficient (**b**, **c**). CAFs cancer-associated fibroblasts, ccRCC clear cell renal cell carcinoma, IHC immunohistochemistry, ISH in situ hybridization, UC urothelial carcinoma.

Given the effects of Am80 administration on TAM polarization in the mT5 and MB49 tumor models, we next examined the infiltration of TAMs and their phenotype in tumor samples surgically resected from patients with ccRCC and UC (Fig. 4). This revealed that the number of Meflin^+^ CAFs significantly correlated with that of CD68^+^ TAMs in both cancer types (Fig. 4a–c). The number of CD163^+^ M2-like TAMs and the ratio of CD163^+^ TAMs to CD68^+^ TAMs were inversely correlated with the number of Meflin^+^ CAFs. These data support the notion that CAF phenotypes are closely associated with TAM phenotypes in tumor tissues, consistent with findings reported by other previous studies [7, 36].

### Requirement of Meflin expression in fibroblasts for Am80-mediated changes in macrophage polarization and anti-PD-L1 therapy efficacy

**Fig. 5:**
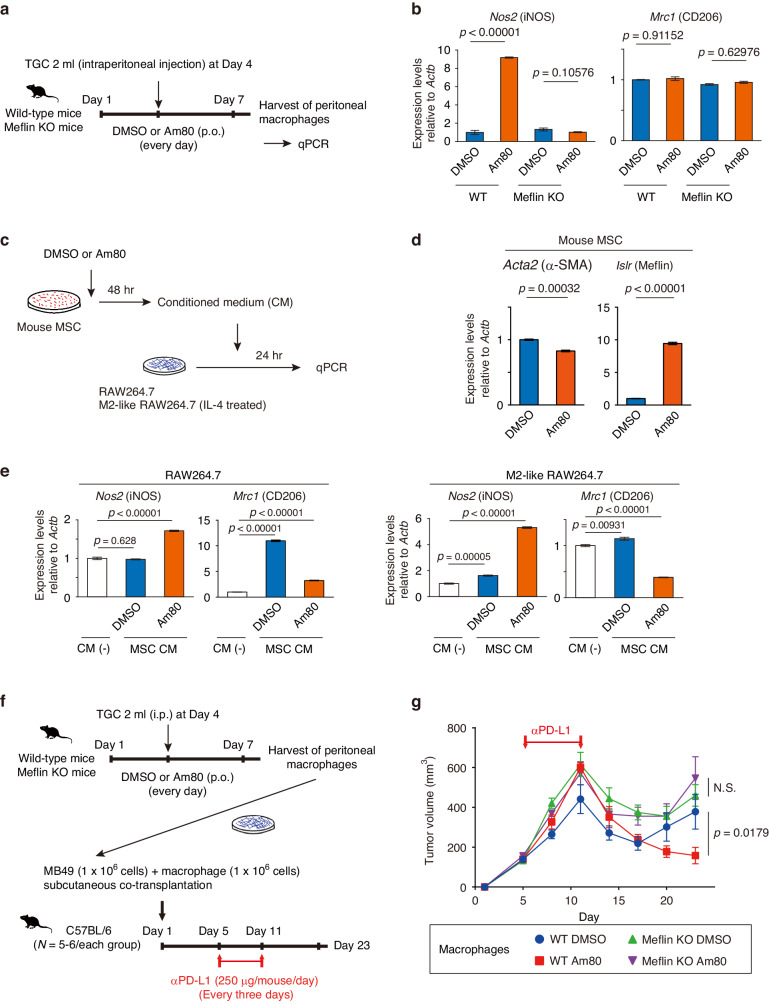
Meflin expression in fibroblasts is required for Am80-mediated macrophage polarization and increases in efficacy of anti-PD-L1 antibody treatment. **a** C57BL/6 wild-type or Meflin KO female mice were orally administered DMSO or Am80 every day from Day 1 to Day 7. The mice were subjected to intraperitoneal injection of 2 ml TGC (3%) on Day 4, followed by isolation of peritoneal macrophages on Day 7, and quantitative PCR (qPCR) for macrophage markers. **b** Expression levels of *Nos2* and *Mrc1* relative to *Actb* in the indicated macrophages as examined by qPCR. **c** Conditioned media (CM) from primary cultured mouse MSCs treated with either DMSO or Am80 (1 µM) for 48 h were added to control RAW264.7 cells or RAW264.7 cells that had been differentiated into M2-type macrophages using recombinant IL-4 (40 ng/ml, 48 h), followed by culture for 24 h and qPCR. **d** Total RNA extracted from MSCs treated with DMSO or Am80 (1 µM) for 48 h was examined for the expressions of *Acta2* (left) and *Islr* (right) relative to *Actb* by qPCR. **e** Expression levels of *Nos2* and *Mrc1* relative to *Actb* in the indicated RAW264.7 cells as examined by qPCR. **f**, **g** Peritoneal macrophages (1 × 10^6^ cells/mouse), isolated from C57BL/6 wild-type or Meflin KO female mice that were administered DMSO or Am80 after intraperitoneal injection of 2 ml 3% TGC, were subcutaneously co-transplanted with MB49 cells (1 × 10^6^ cells/mouse) into wild-type mice on Day 1, followed by anti-PD-L1 therapy from Day 5 to Day 11 (**f**), and measurement of the volume of the developed tumors (**g**). Statistical differences were assessed with 1-way ANOVA with the Tukey test (**b**), Student *t*-test (**d**, **g**), and 1-way ANOVA with the Dunnett test (**e**). KO knockout, MSCs mesenchymal stem cells, NS not significant, qPCR quantitative polymerase chain reaction, PD-L1 programmed cell death ligand 1, TGC thioglycollate.

To address how Meflin expression in CAFs is involved in Am80-mediated changes in macrophage polarization in tumors, we harvested thioglycollate (TGC)-elicited peritoneal macrophages from WT and Meflin KO mice after administration of Am80, followed by quantitative PCR (qPCR) to examine the expression levels of macrophage marker genes (Fig. 5a). We found that *Nos2*, but not *Mrc1*, was highly upregulated in macrophages isolated from Am80-administered WT mice but not Meflin KO mice (Fig. 5b). This finding was supported by an experiment in which we used cultured MSCs and a macrophage cell line RAW264.7 polarized to the M2-like phenotype by culturing cells with recombinant IL-4 (Fig. 5c). In this experiment, we used conditioned medium (CM) derived from cultures with control MSCs or MSCs pretreated with Am80 that exhibited a significant increase in Meflin expression (Fig. 5c, d). Interestingly, CM prepared from control MSCs, but not when pretreated with Am80, induced the polarization of RAW264.7 to *Mrc1*^+^ M2-like cells (Fig. 5e, left panel). In contrast, CM from MSCs treated with Am80 significantly increased the expression of *Nos2*, whereas it downregulated *Mrc1* expression in M2-like RAW264.7 cells (Fig. 5e, right panel). Together, these data suggest that Am80-induced changes in the MSC state – including the upregulation of Meflin expression – dictate macrophages to polarize to an M1-like phenotype.

Next, we isolated macrophages from either WT and Meflin KO mice that were treated with dimethylsulfoxide (DMSO) or Am80, and co-implanted these macrophages with MB49 cells in C57BL/6 mice. These mice were subsequently treated with anti-PD-L1 antibodies, and tumor size was measured (Fig. 5f). This revealed that macrophages isolated from WT mice that were administered Am80, but not those from mice in the other groups, were able to produce durable anti-MB49 tumor responses after anti-PD-L1 treatment (Fig. 5g). These data support the notion that Am80 administration skews macrophages toward the M1-like phenotype in response to Am80-induced increases in Meflin expression in CAFs, which is partly involved in the induction of durable responses against MB49 tumors in response to anti-PD-L1 antibody treatment.

### Am80 induces the expression of *Rarres2* encoding chemerin in Meflin^+^ CAFs

**Fig. 6:**
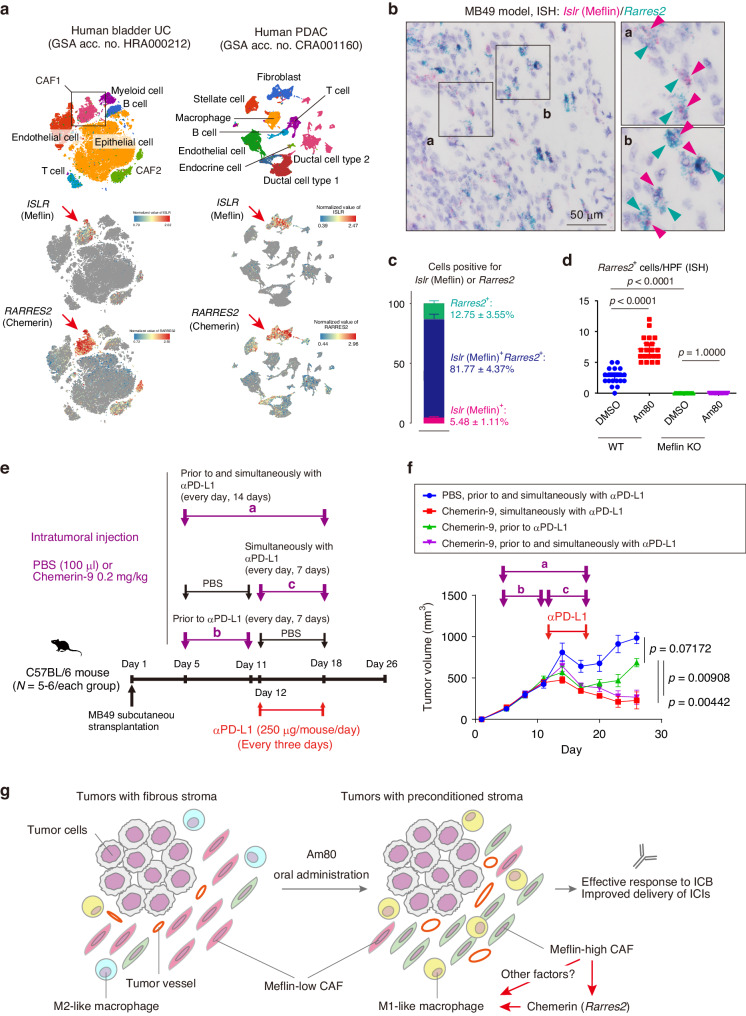
Expression of chemerin in Meflin^+^ CAFs and its role in response to anti-PD-L1 therapy in the MB49 model. **a** Publicly available datasets of single-cell RNA transcriptomic analysis of human bladder UC (left) and PDAC (right) were analyzed for the expression of *ISLR* and *RARRES2*, which encode Meflin and chemerin, respectively. Shown are uniform manifold approximation and projection visualization of transcriptomes of all cells depicted by BBrowser. Arrows denote the fibroblast clusters that co-express *ISLR* and *RARRES2*. **b**, **c** Selective expression of *Rarres2* in Meflin^+^ CAFs. Tumor sections from the MB49 model were double stained for *Islr* and *Rarres2* by ISH (**b**). Boxed areas (a, b) are magnified in adjacent panels. Magenta and cyan arrowheads denote *Islr* and *Rarres2* signals, respectively. The numbers of cells single- or double-positive for *Islr* and *Rarres2* were counted and quantified (**c**). **d** Number of *Rarres2*^+^ cells detected in tissue sections prepared from MB49 tumors developed in wild-type and Meflin KO mice that were administered DMSO or Am80 (the experiment shown in Fig. 3a) were counted, followed by quantification. **e**, **f** C57BL/6 wild-type or Meflin KO female mice were subcutaneously transplanted with MB49 cells (1 × 10^6^ cells/mouse), followed by intratumoral injection of PBS or recombinant chemerin-9 (0.2 mg/kg) during the indicated periods, and intraperitoneal injection of anti-PD-L1 antibody (**e**). Tumor volumes of the developed tumors were measured over time (**f**). **g** Schematic diagram showing a working hypothesis for the mechanism of Am80-mediated tumor sensitization to ICB therapy. The mechanisms by which Meflin induces the expression of chemerin and how it is involved in the induction of M1-like macrophages remain unaddressed in the present study. Statistically significance was assessed using 1-way ANOVA with the Tukey test (**d**, **f**). HPF high-power field (×40 objective lens), ICB immune checkpoint blockade, ISH in situ hybridization, KO knockout, PDAC pancreatic ductal adenocarcinoma, PD-L1 programmed cell death ligand 1, UC urothelial carcinoma.

To examine the possibility that Meflin directly regulates macrophage function, we next added CM of CHO cells that stably express mouse Meflin (mMeflin) to RAW264.7 cells polarized to the M2-like phenotype (Supplementary Fig. S11A). We found, however, no apparent effect of Meflin on the phenotype of M2-like RAW264.7 cells (Supplementary Fig. S11B). We have therefore focused on the retinoic acid receptor responder 2 (*Rarres2*) gene that encodes chemerin, which was identified as one of the genes tightly co-expressed and co-regulated with Meflin when we first reported Meflin as a fibroblast marker [18]. Chemerin is a cytokine that possesses versatile biological functions, including the regulation of metabolism, angiogenesis, and the innate and adaptive immune system, where it serves as a chemoattractant for several immune cell types [37–39]. The analysis of a publicly available dataset of scRNA-seq obtained from human UC and PDAC samples [30, 40], as well as double ISH for *Rarres2* and *Islr* on tissue sections from MB49 tumors and human PDAC, showed their co-expression in CAFs (Fig. 6a–c, Supplementary Fig. S12A–C). MB49 tumors developed in Am80-administered WT mice but not Meflin KO mice exhibited a significant infiltration of *Rarres2*^+^ cells (Fig. 6d). This suggests that Meflin is required for Am80-mediated induction of *Rarres2* expression in CAFs.

### Chemerin plays a role in accelerating the efficacy of anti-PD-L1 antibody treatment in the MB49 model

We next addressed the role of chemerin expression in CAFs in anti-PD-L1 therapy. To this end, we intratumorally administered chemerin-9, a nanopeptide corresponding to the C-terminus of processed chemerin that retains the activity of full-size chemerin [41], to the MB49 model at various schedules together with anti-PD-L1 antibodies (Fig. 6e). We observed sustained efficacy of anti-PD-L1 therapy when chemerin-9 was administered simultaneously with, but not prior to, anti-PD-L1 therapy (Fig. 6f). Chemerin-9 administration prior to anti-PD-L1 antibody treatment transiently augmented the tumor-suppressive effects of anti-PD-L1 treatment. However, this effect disappeared over time, possibly due to the short half-life of the recombinant chemerin-9 [42].

IHC for various immune cell markers showed that chemerin-9 induced variable alterations in the infiltration of several types of immune cells, which differed between WT and Meflin KO mice (Supplementary Fig. S13A-D). The infiltration of CD11b^+^ or Ly6G^+^ myeloid cells and CD86^+^ M1-like TAMs, but not T cells, was significantly altered by intratumoral injection of chemerin-9 to the same extent as Am80 (Supplementary Fig. S13C, D). This was consistent with previous studies that described the actions of chemerin on various inflammatory reactions through its receptors, chemokine-like receptor-1 and G-protein coupled receptor 1 [37]. Am80-mediated decreases in the numbers of CD163^+^ M2-like TAMs and PD1^+^ T cells were also reproduced by chemerin-9 in tumors developed in WT mice but not Meflin KO mice (Supplementary Fig. S13D, E). In contrast, Am80-mediated changes in PD-L1^+^ cells, which mainly comprise TAMs because MB49 cells are mostly negative for PD-L1 (Supplementary Fig. S8), were not reproduced by chemerin-9 (Supplementary Fig. S13E), suggesting that chemerin partly mediates the effects of Am80 in the MB49 model.

Finally, the role of Meflin expression in CAFs in chemerin-mediated TAM polarization was examined by using peritoneal macrophages isolated from WT and Meflin KO mice which were i.p. administered with chemerin-9 (Supplementary Fig. S14A). We found that both the M1- and M2-like phenotypes of macrophages were activated by chemerin-9, irrespective of whether they were isolated from WT or Meflin KO mice (Supplementary Fig. S14B). This seemingly contradictory result from our findings in the MB49 model (Supplementary Fig. S13D) was further investigated by treating control RAW264.7 cells or those polarized to the M2-like phenotype (M2 RAW264.7) with chemerin-9 (Supplementary Fig. S14C). Unexpectedly, chemerin-9 at either 0.1 µM or 0.1 nM significantly downregulated the expression of *Nos2* and *Mrc1* in both types of RAW264.7 cells, suggesting that recombinant chemerin-9 by itself is not sufficient to induce macrophage polarization in culture (Supplementary Fig. S14D). The data obtained by the mouse model and cultured macrophages experiments suggest that chemerin may mediate TAM polarization through an indirect pathway involving tumor cells and Meflin^+^ CAFs.

## Discussion

In the present study, we show that Am80-mediated induction of Meflin expression in CAFs primes tumors to respond to anti-PD-L1 therapy in murine UC and PDAC models (Fig. 6g). We determined the optimal timing for Am80 administration as prior to, but not concomitant with, anti-PD-L1 therapy. Am80-induced Meflin expression in CAFs was accompanied by robust changes in the infiltration and phenotype of immune cells, including the polarization of TAMs toward the M1-like phenotype that is known to associate with favorable tumor response to ICB therapy [43]. We also showed that Meflin regulates chemerin expression in CAFs treated with Am80, which then induces TAM polarization. However, recombinant chemerin-9 did not directly affect macrophage polarization, implying complex multifactorial interactions between CAFs and TAMs. The clinical relevance of these findings was also supported by the analysis of patients with UC and ccRCC who received ICB therapy, consistent with our previous study in NSCLC [28]. Taken together, these findings support the notion that manipulating CAF phenotypes before treatment may be a strategy to improve the efficacy of ICB therapy in clinical settings.

Consistent with our previous study [24], we showed that Am80-mediated sensitization of tumors to anti-PD-L1 antibodies could be partially explained by improvements in intratumoral delivery of the injected antibodies. However, this could not fully explain why simultaneous administration of Am80 had no significant effect on the efficacy of anti-PD-L1 treatment. Given that previous studies have shown that Am80 has the potential to inhibit the differentiation of naïve T cells to helper Th17 cells and induction of cytotoxic CD8^+^ and regulatory T cells in several disease mouse models [44–46], we speculated that Am80 might abrogate immune responses requisite for ICB response. However, continued Am80 administration prior to and alongside anti-PD-L1 antibody treatment was effective in improving its efficacy, posing the question of whether Am80 exerts an immune suppressive role in this in vivo context. Importantly, Am80 administration induced the polarization of TAMs into an M1-like phenotype, consistent with previous studies [46]. Our study went a step further and demonstrated that chemerin produced by Meflin^+^ CAFs might be responsible for this TAM polarization. Unfortunately, intratumoral injection of chemerin-9 at a dose of 0.2 mg/kg prior to anti-PD-L1 antibody treatment failed to completely recapitulate the effect of Am80 administration, which may be due to the rapid degradation and short half-life (~30 min) of chemerin-9 [42]. Therefore, the exact mode of action of full-length chemerin on the response to ICB therapy remains to be elucidated by further research.

Another intriguing but unexpected finding in the present study was that Meflin KO mice failed to reproduce the effects of Am80 on ICB therapy and the alterations of the TIME observed in WT mice. Given the specific expression of Meflin in fibroblasts [17, 18, 21–23], the data suggested that CAFs act upstream of or along with immune cells in determining response to ICB therapy. This is in agreement with a recent study that showed that the genetic depletion of CAFs expressing Leucine-rich repeat containing 15 (LRRC15) increased the relative proportion of normal fibroblasts expressing Peptidase inhibitor 16 (Pi16) and dermatopontin (Dpt), which optimized the TIME, resulting in high sensitivity to ICB therapy [29]. We previously showed that Meflin expression is highly enriched in normal tissue-resident fibroblasts or undifferentiated MSCs, and its expression undergoes rapid downregulation during the differentiation into myofibroblasts, myCAFs, and mesenchymal lineage cells such as osteoblasts, chondrocytes, and adipocytes [17–19, 21]. Thus, an important issue that remains to be addressed is how normal fibroblasts expressing Pi16, Dpt, and Meflin govern the immune microenvironment and counteract with myofibroblasts and myCAFs in normal tissues and other disease contexts.

The idea of reprogramming CAFs to improve the efficacy of cancer therapeutics has previously been proposed, and some methods have already moved into clinical trials [3, 4, 8, 10, 47, 48]. These include studies that assess the impact of ATRA and vitamin D on the efficacy of conventional chemotherapeutics and ICB therapies. We have also started an investigator-initiated clinical trial to determine the therapeutic efficacy of the Am80 and gemcitabine/nab-paclitaxel combination therapy in patients with unresectable advanced PDAC [49]. The results of these clinical trials should provide important insights into the significance of CAF heterogeneity in response to chemotherapies and ICB therapy in human cancer.

## Materials and methods

### Human tissue samples

We retrospectively enrolled a cohort of patients with ccRCC and UC at Nagoya University Hospital, Kariya Toyota General Hospital, and Japan Community Healthcare Organization Chukyo Hospital to identify the involvement of Meflin^+^ CAFs in clinical response to ICB therapy and their clinical outcomes.

### Clinical efficacy analysis

OS was defined as the time from the start of ICB therapy until death from any cause. PFS was defined as the time from ICB therapy initiation until disease progression or death from any cause. Patient follow-up ended when an outcome was recorded or censored as of the database lock on 31 December 2022. Response to ICB therapy was determined according to immunotherapy Response Evaluation Criteria in Solid Tumors (iRECIST) at each time point.

### Animals

The generation of Meflin KO mice was described previously [17, 18, 21–24, 28]. Genomic DNA extracted from mouse tails was used for polymerase chain reaction (PCR)-based genotyping of WT and Meflin KO mice. The sequences of the primers used for genotyping of the Meflin (*Islr*) gene were as follows: PCR1 forward, 5’-GCTGCATTTGAGCTGAGCCTCTGG-3’; PCR1 reverse, 5’-AACCCCTTCCTCCTACATAGTTGG-3’; PCR2 forward, 5’-TGAGGTTAGCCTGGGGACTTCAC-3’; PCR2 reverse, 5’-GGCTAGAACTCTCAAAGTAGGTCAGG-3’.

### Tumor immunotherapy

To investigate the efficacy of ICB therapy in subcutaneous tumor models, anti-mouse PD-L1 (clone 10 F.9G2, rat IgG2b, RRID: AB_2800599; BioLegend, 250 mg per mouse per injection), anti-mouse PD-1 (clone RMP1-14, rat IgG2a, RRID: AB_2800578; BioLegend, 200 mg per mouse per injection), and isotype control (RTK4530 and RTK2758; BioLegend) antibodies were administered intraperitoneally to mice at the indicated regimens.

### Reagents for animal studies

Concentrated stock solutions of 20 mg/ml Am80 (cat. no. 3507; Tocris) in DMSO and those of 1 mg/ml chemerin-9 (cat. no. RP20248; GenScript) in PBS were stored protected from light, for up to 1 month. The Am80 stock solutions and DMSO (controls) were diluted in the same volume of 0.5% carboxymethylcellulose (CMC) solution (Wako Chemicals, Osaka, Japan) to the appropriate dose before oral administration.

### Syngeneic tumor studies

In vivo tumor studies were performed as follows: 6-week-old C57BL/6 WT and Meflin KO female mice were subcutaneously transplanted in the right flank with 1 × 10^6^ cells suspended in 100 μl PBS. Tumor sizes were measured on the indicated days with calipers, and tumor volumes were calculated using the formula X^2^ × Y × 0.5, where X is the smaller diameter and Y is the larger diameter.

### Culture of cell lines

The mouse PDAC cell line mT5 was generously provided by David Tuveson (Cold Spring Harbor Laboratory) and Chang-Il Hwang (UC Davis College of Biological Sciences, Davis, CA). The mouse UC cell line MB49 was purchased from Merck Inc (cat. no. SCC148). The mouse gastric cancer cell line YTN5 was generated as previously described [50]. The mouse lung cancer cell line Ex-3LL/CMV-Luc#1 was obtained from the Japanese Collection of Research Bioresources (JCRB). The mouse macrophage cell line RAW264.7 was purchased from ATCC (cat. no. TIB-71). All cell lines were authenticated by routine morphological and growth analyses and routinely screened for *Mycoplasma* contamination using 4’,6-diamidino-2-phenylindole staining.

### Thioglycollate (TGC)-elicited peritoneal macrophages

TGC-elicited peritoneal macrophages were isolated from 6-7 weeks-aged C57BL/6 WT or Meflin KO female mice. The mice received oral administration of DMSO or Am80 or were intraperitoneally (i.p) injected with PBS or chemerin-9 (0.2 mg/kg) every day from Day 1 to Day 7, followed by injection of 2 ml 3% TGC broth (Cat. no. 5601; Nissui Pharmaceutical) into the peritoneal cavity of each mouse. On Day 7, the mice were sacrificed, followed by injection of 6 ml cold PBS into the peritoneal cavity, gentle massage of the abdomen, and aspiration of PBS containing peritoneal macrophages. Cells were collected by centrifugation (1,000 rpm, 3 min) and washed twice. Red blood cells were discarded using Red Blood Cell Lysis Solution (Miltenyi Biotec, cat. no. 130-094-183), after which residual cells were collected (1,000 rpm, 3 min) and washed twice.

### Co-transplantation model

In the co-transplantation models, TGC-elicited peritoneal macrophages (1 × 10^6^ cells/mouse) were isolated from C57BL/6 WT or Meflin KO female mice that were pretreated with DMSO or Am80. Isolated cells were subcutaneously co-transplanted with MB49 cells (1 × 10^6^ cells/mouse) in the right flanks of 6-week-old C57BL/6 WT female mice. Tumor sizes were measured on the indicated days with calipers, and tumor volumes were calculated using the formula X^2^ × Y × 0.5, where X is the smaller diameter and Y is the larger diameter.

### Statistical analyses

We used GraphPad Prism 7 or R v.4.0.5 for statistical analysis. Patient characteristics and binary outcomes were compared between two groups using the Fisher’s exact test or the Mann-Whitney U test. Survival was analyzed using the Kaplan-Meier approach and differences in survival were analyzed using the log-rank (Mantel-Cox) test. The relationships between groups were compared using Student *t*-test unless otherwise specified. For multiple testing, 1-way ANOVA with the Tukey test or Dunnett test was employed. Quantitative interrelationships were determined using the Spearman’s rank-order correlation coefficient. Statistical significance was set at *p* < 0.05.

Detailed protocols for animal experiments, histology, cell biology, biochemistry, PCR, and single-cell RNA sequencing data analysis are described in the Supplementary Data.

### Supplementary information

Supplementary Information

## Data Availability

Data are available upon reasonable request. All data relevant to the study are included in the article or uploaded as supplementary information. Data are available upon reasonable request.
