# Supplementary material for: Synthetic retinoid-mediated preconditioning of cancer-associated fibroblasts and macrophages improves cancer response to immune checkpoint blockade
Source: Br J Cancer. 2024 Jun 7;131(2):372–86. doi: 10.1038/s41416-024-02734-3 (PMC11263587; doi:10.1038/s41416-024-02734-3)
Supplement: Supplementary file 1 — Supplementary Information [file 41416_2024_2734_MOESM1_ESM.pdf]

Synthetic retinoid-mediated preconditioning of cancer-associated fibroblasts and macrophages improves cancer response to immune checkpoint blockade

Owaki *et al.*

Supplementary Data

1. Supplementary Table S1-S3

2. Supplementary Figures S1-S14

3. Supplementary Methods

Supplementary Table S1. Hazard ratios and P values for multivariate Cox proportional hazard regression model analysis in patients with ccRCC and UC who did not receive ICB therapy.

(a) Hazard ratios and P values for multivariate Cox proportional hazard regression model analysis in patients with ccRCC who did not receive ICB therapy

Variable	Hazard ratio (95% CI) for OS	P value for OS	Hazard ratio (95% CI) for PFS	P value for PFS
Age at surgery		0.255		0.336
≤65	Reference		Reference	
65<	2.3840 (0.5349 - 10.620)		1.708 (0.57420 - 5.079)	
Sex		0.917		0.854
Male	Reference		Reference	
Female	1.0780 (0.2598 - 4.476)		1.108 (0.37370 - 3.283)	
TNM stage		<0.001		0.005
≤3	Reference		Reference	
4	13.3100 (2.8920 - 61.300)		8.318 (1.87000 - 37.010)	
ECOG-PS		0.667		0.357
0 or 1 (Good)	Reference		Reference	
2≤ (Poor)	0.6619 (0.1013 - 4.325)		0.315 (0.02701 - 3.673)	
Meflin Expression		0.673		0.110
Low	Reference		Reference	
High	1.3500 (0.3350 - 5.436)		2.484 (0.81390 - 7.580)	

(b) Hazard ratios and P values for multivariate Cox proportional hazard regression model analysis in patients with UC who did not receive ICB therapy

Variable	Hazard ratio (95% CI) for OS	P value for OS	Hazard ratio (95% CI) for PFS	P value for PFS
Age at surgery		0.128		0.131
≤65	Reference		Reference	
65<	2.0540 (0.81350 - 5.1840)		1.7750 (0.8432 - 3.737)	
Sex		0.819		0.539
Male	Reference		Reference	
Female	1.1540 (0.33830 - 3.9390)		1.3770 (0.4962 - 3.823)	
Primary site		0.021		0.931
Bladder	Reference		Reference	

Upper urinary tract	0.3754 (0.16320 - 0.8638)		0.9673 (0.4586 - 2.040)	
TNM stage		0.220		0.380
0 or 1	Reference		Reference	
2≤	2.0180 (0.65720 - 6.1960)		1.4390 (0.6384 - 3.243)	
Neoadjuvant chemotherapy		0.177		0.029
No	Reference		Reference	
Yes	1.9320 (0.74320 - 5.0230)		2.5460 (1.1030 - 5.877)	
Brinkman index		0.874		0.850
<400	Reference		Reference	
400≤	1.1050 (0.32330 - 3.7750)		1.1020 (0.4044 - 3.003)	
ECOG-PS		0.272		0.655
0 or 1 (Good)	Reference		Reference	
2≤ (Poor)	0.4020 (0.07917 - 2.0410)		0.7449 (0.2050 - 2.707)	
Meflin Expression		0.010		0.028
Low	Reference		Reference	
High	3.3250 (1.33000 - 8.3140)		2.2690 (1.0920 - 4.714)	

Supplementary Table S2. Characteristics of patients with ccRCC and UC who did not receive ICB therapy.

(a) Characteristics of patients with ccRCC who did not receive ICB therapy

	Meflin expression		P value
Variable	High	Low	
Median age (year) [range]	69 [47-79]	64 [39-80]	0.392
Sex			1
Male	14	18	
Female	8	9	
TNM stage			0.0849
1	4	14	
2	4	2	
3	10	9	
4	4	2	
ECOG-PS			0.581
2≤ (Poor)	2	1	
0 or 1 (Good)	20	26	

(b) Characteristics of patients with UC who did not receive ICB therapy

	Meflin expression		P value
Variable	High	Low	
Median age (year) [range]	71 (55-88)	67 (36-85)	0.249
Sex			1
Male	16	17	
Female	13	13	
Primary site			0.789
Upper urinary tract	18	20	
Bladder	11	10	
TNM stage			0.0896
0 or 1	6	10	
2	3	9	
3	15	8	
4	5	3	
Neoadjuvant chemotherapy			0.333
No	22	26	
Yes	7	4	
Brinkman index			1
<400	14	14	
400≤	15	16	
ECOG-PS			0.353
2≤ (Poor)	26	29	
0 or 1 (Good)	3	1	

Supplementary Table S3. Characteristics of patients with ccRCC and UC treated with ICB therapy.

(a) Characteristics of patients with ccRCC who received ICB therapy

	Meflin expression		P value
Variable	High	Low	
Median age (year) [range]	63 [40-85]	64 [67-78]	0.371
Sex			0.0704
Male	22	13	
Female	1	5	
TNM stage			0.172
1	1	5	
2	1	0	
3	7	4	
4	14	9	
IMDC criteria			0.38
Favorable	3	3	
Intermediate	19	12	
Poor	1	3	
ECOG-PS			1
2≤ (Poor)	1	1	
0 or 1 (Good)	22	17	
Treatment line			0.856
1st	9	6	
2nd	7	5	
3rd	7	7	

(b) Characteristics of patients with UC who received ICB therapy

	Meflin expression		P value
Variable	High	Low	
Median age (year) [range]	72 [42-87]	70.5 [49-88]	0.944
Sex			1
Male	19	16	
Female	8	6	
Primary site			0.571
Upper urinary tract	15	10	
Bladder	12	12	
TNM stage			0.0524
0 or 1	8	1	
2	4	4	
3	12	9	
4	3	8	
Neoadjuvant chemotherapy			0.0268
No	23	12	
Yes	4	10	
Brinkman index			0.777
<400	11	8	
400≤	16	14	

ECOG-PS			0.266
2≤ (Poor)	3	6	
0 or 1 (Good)	24	16	

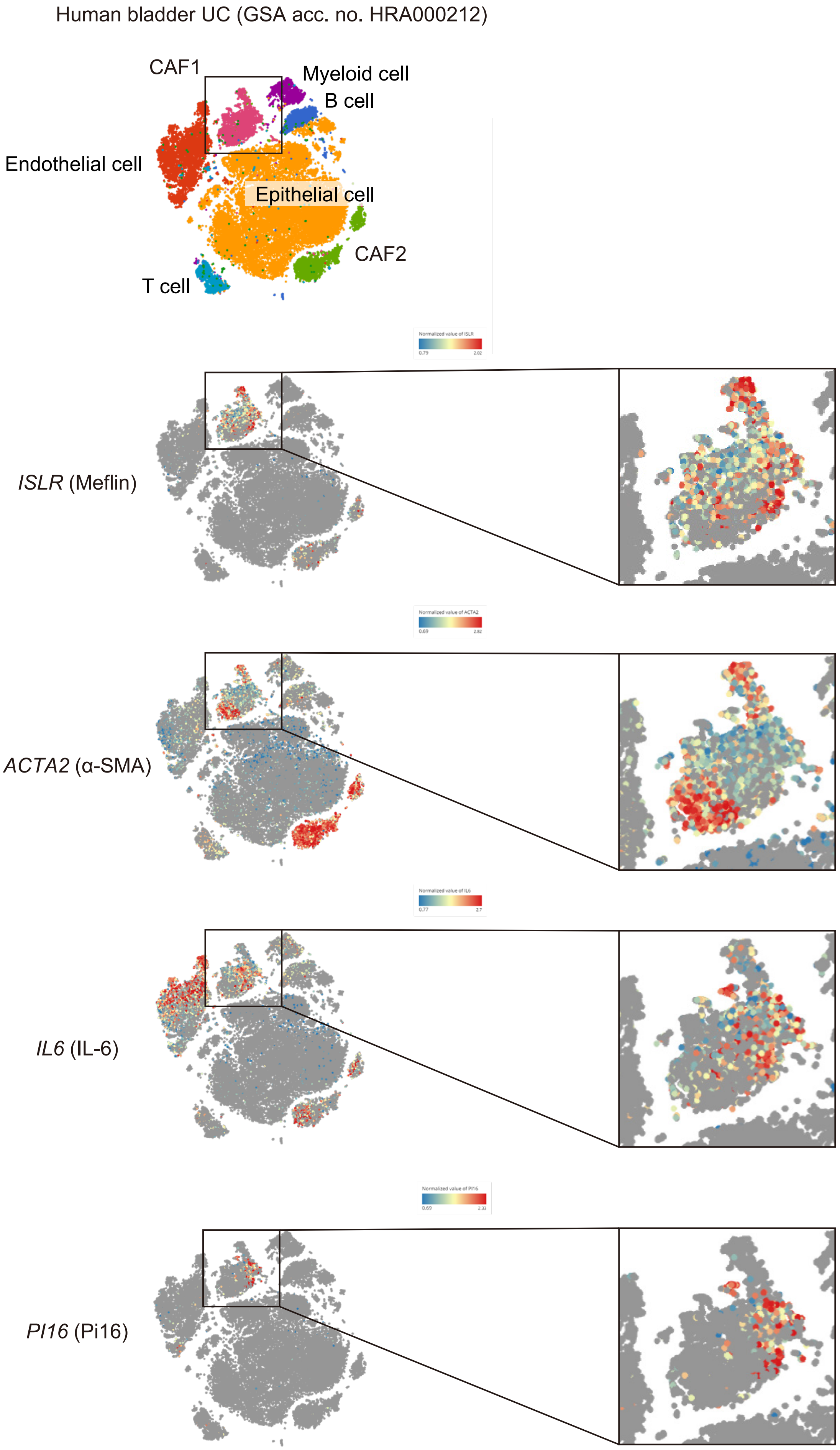

Supplementary Figure S1

Supplementary Fig. S1. Expression of *ISLR* in a distinct CAF subset that preferentially expresses *ACTA2*.

A publicly available single-cell RNA transcriptomic dataset of human bladder UC was analyzed for the expression of *ISLR* (Meflin), *ACTA2* (α-SMA), *IL6* (IL-6), and *PI16* (Pi16). CAF clusters were magnified in adjacent panels, showing that *ISLR* and *ACTA2* are expressed in different subsets within the CAF cluster. *CAF: cancer-associated fibroblast.*

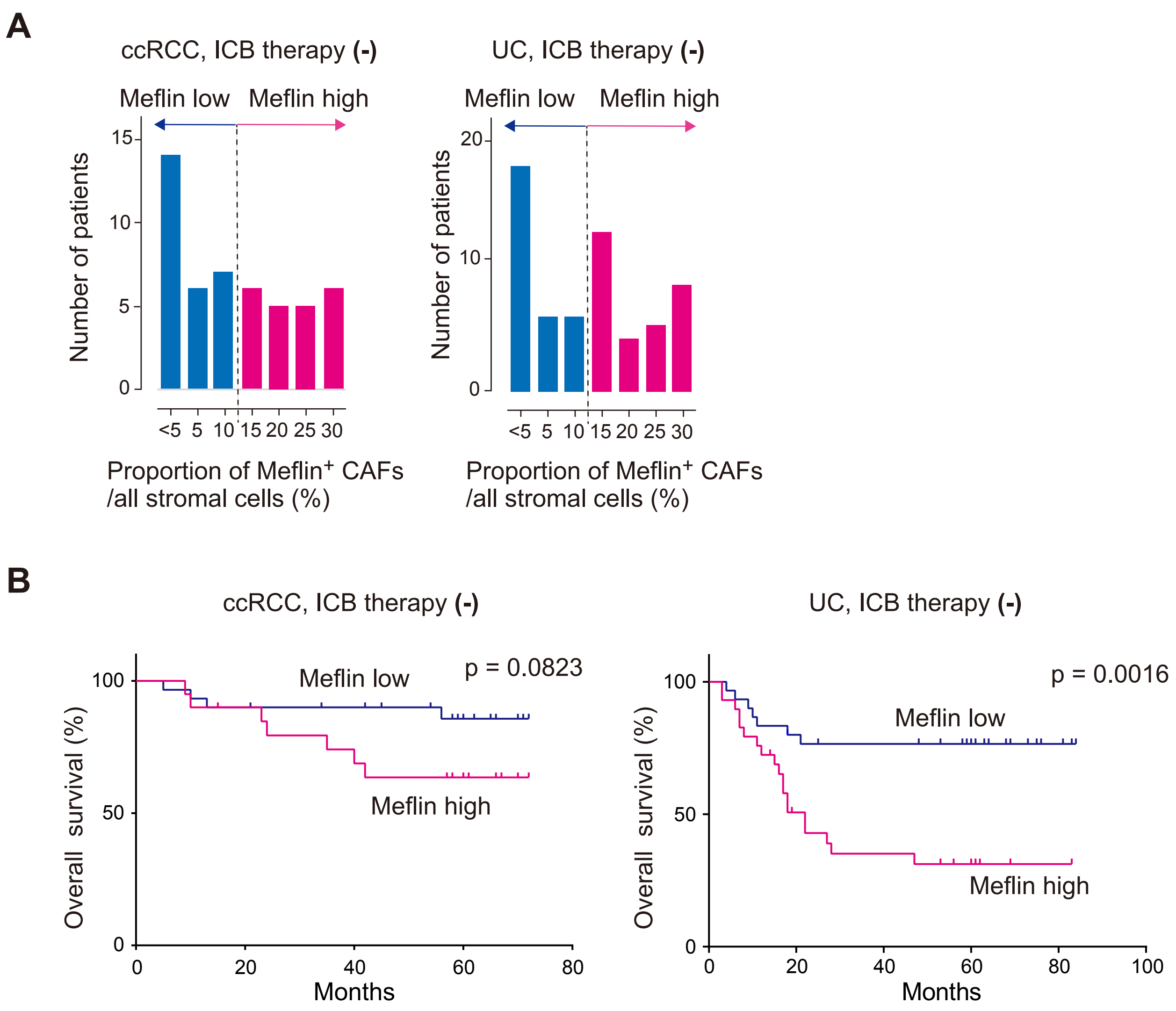

Supplementary Figure S2

Supplementary Fig. S2. Meflin expression is correlated with poor outcomes in patients with ccRCC or UC who did not receive ICB therapy.

(A) Patients with ccRCC (left) or UC (right) not treated with ICB therapy were stratified by the numbers of Meflin$^+$ CAFs in all stromal cells.

(B) Overall survival of Meflin-high and -low ccRCC (left) or UC (right) cases who were not treated with ICB therapy.

Differences between groups were assessed with the log-rank (Mantel-Cox) test (**B**). *CAF: cancer-associated fibroblast; ccRCC: clear cell renal cell carcinoma; ICB: immune checkpoint blockade; UC: urothelial carcinoma.*

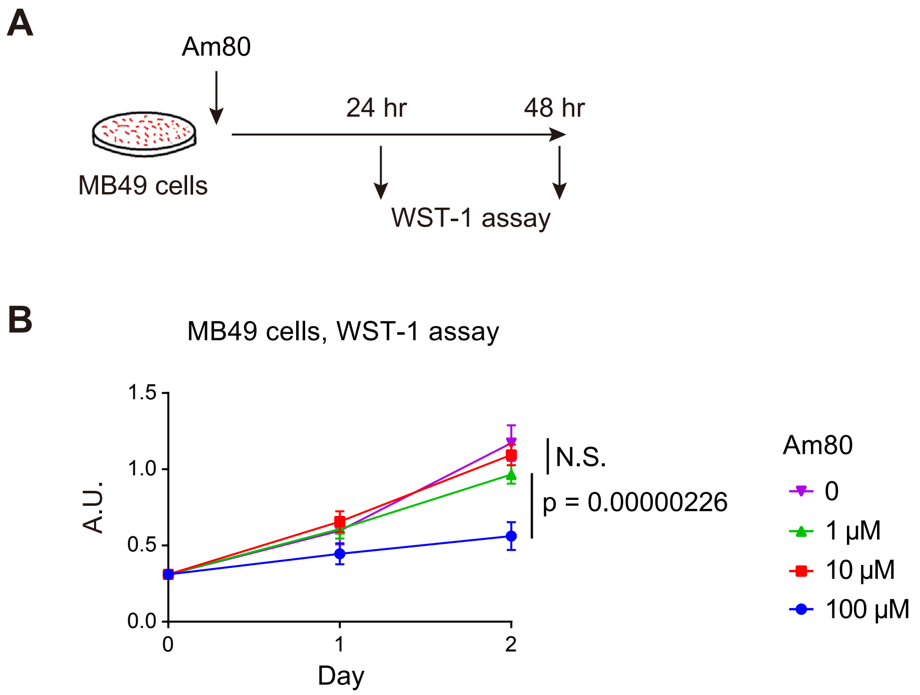

Supplementary Figure S3

Supplementary Fig. S3. Marginal effects of Am80 on the proliferation of MB49 cells *in vitro*.

(A) MB49 cells (1×10^3 cells) were plated in wells of a 96-well plate and cultured in a medium containing 10% FBS, followed by treatment with Am80 (1, 10, 100 μM) or DMSO. After treatment, cells were assessed with the WST-1 assay.

(B) Proliferation of MB49 cells was evaluated each day using WST-1 assays, followed by quantification.

The statistical methods used are 1-way ANOVA with the Tukey test (**B**). *FBS: fetal bovine serum*.

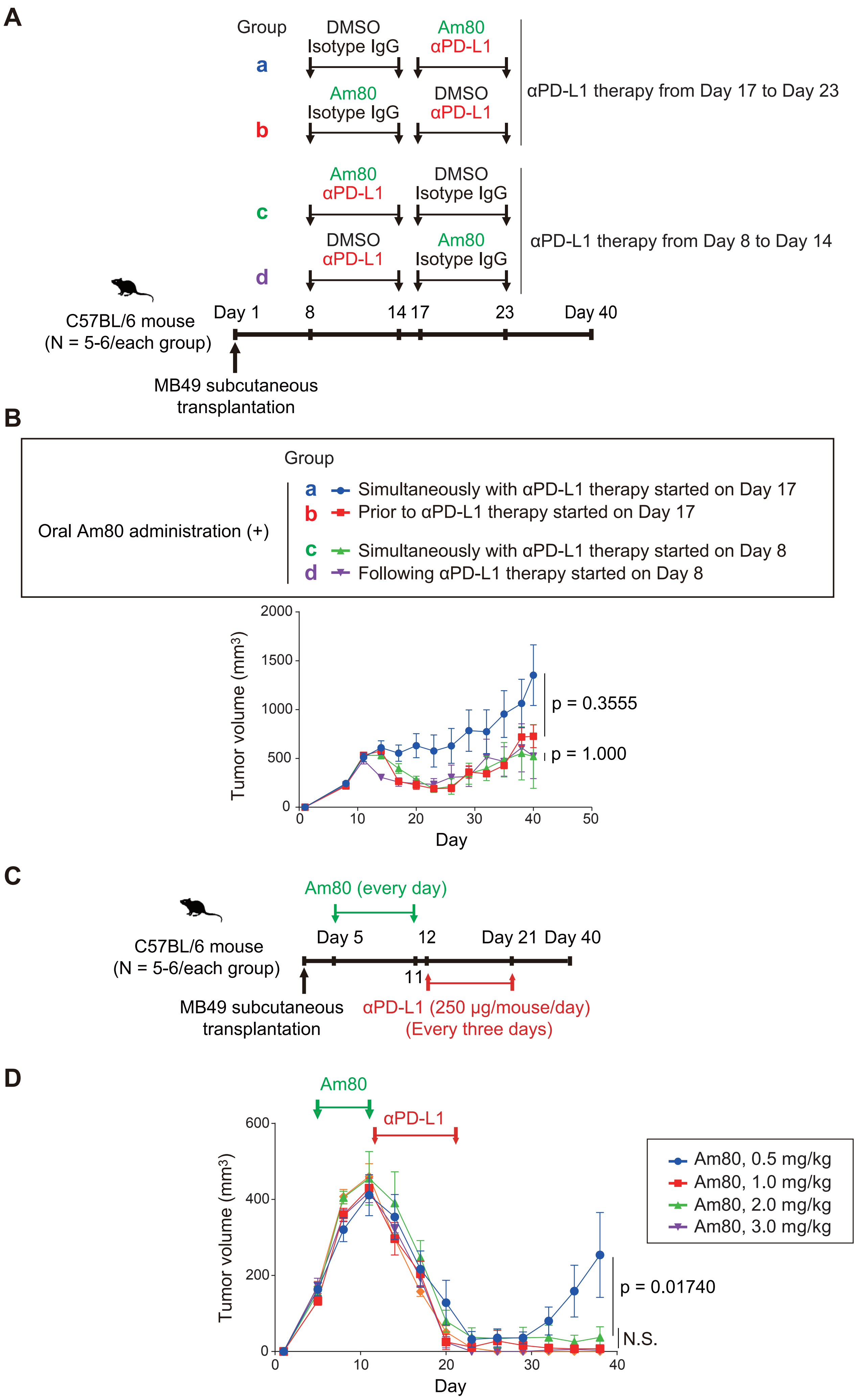

Supplementary Figure S4

Supplementary Fig. S4. A priori oral Am80 administration enhances the efficacy of anti-PD-L1 antibody treatment in the MB49 UC mouse models.

(A) C57BL/6 wild-type female mice were subcutaneously transplanted with MB49 cells (1×10^6 cells/mouse), followed by oral administration of either Am80 (2.0 mg/kg) or DMSO and intraperitoneal injection of anti-PD-L1 antibodies at the indicated schedules.

(B) Time courses of the volume of tumors developed in mice treated by the indicated regimens.

(C) C57BL/6 wild-type female mice were subcutaneously transplanted with MB49 cells (1×10^6 cells/mouse), followed by oral administration of Am80 at the indicated doses (0.5, 1.0, 2.0, and 3.0 mg/kg) prior to intraperitoneal injection of anti-PD-L1 antibodies.

(D) Time courses of the volumes of tumors of the indicated groups.

Differences between groups were analyzed using 1-way ANOVA with the Tukey test (**B** and **D**). *PD-L1: programmed cell death ligand 1.*Statistical analysis was performed using 1-way ANOVA with Tukey test (**B** and **D**).

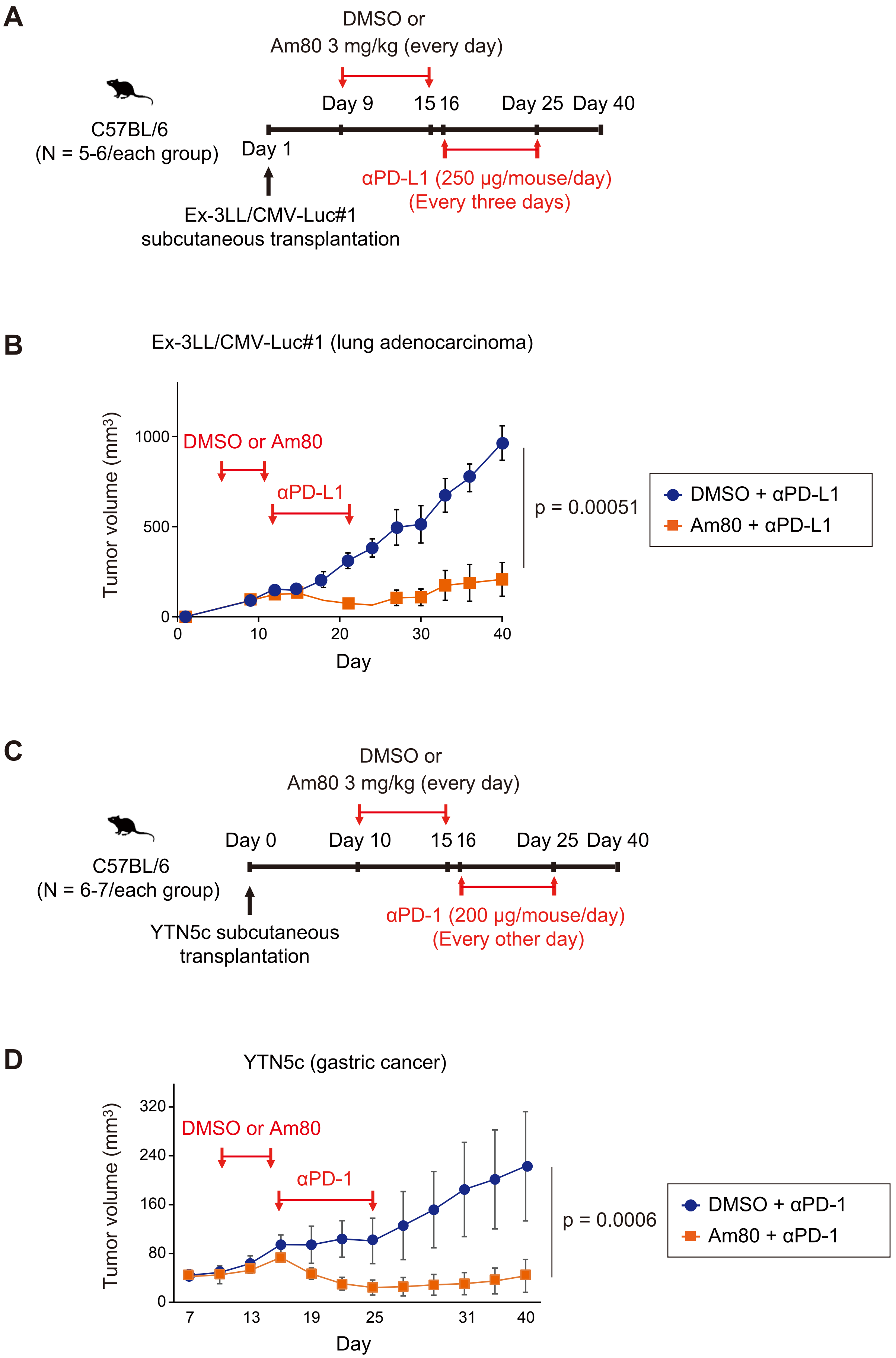

Supplementary Figure S5

Supplementary Fig. S5. Effect of oral Am80 administration on the efficacy of ICB therapy in mouse models of lung and gastric cancers.

(A, B) C57BL/6 wild-type female mice were subcutaneously transplanted with Ex-3LL/CMV-Luc#1, a derivative of LLC (Lewis Lung Carcinoma) cells transduced with Luciferase (1×10^6 cells/mouse), followed by oral administration of DMSO or Am80 and subsequent intraperitoneal injection of anti-PD-L1 antibodies **(A)** and the measurement of tumor volume **(B)**.

(C, D) C57BL/6 wild-type female mice were subcutaneously transplanted with YTN5c cells (5×10^6 cells/mouse), which is a subline of the mouse gastric cancer cell line YTN5, followed by oral administration of DMSO or Am80 and subsequent intraperitoneal injection of anti-PD-1 antibodies **(C)** and the measurement of tumor volume **(D)**.

Differences were assessed using the Welch's t-test (**B** and **D**). *PD-1: programmed cell death protein 1; PD-L1: programmed cell death ligand 1.*

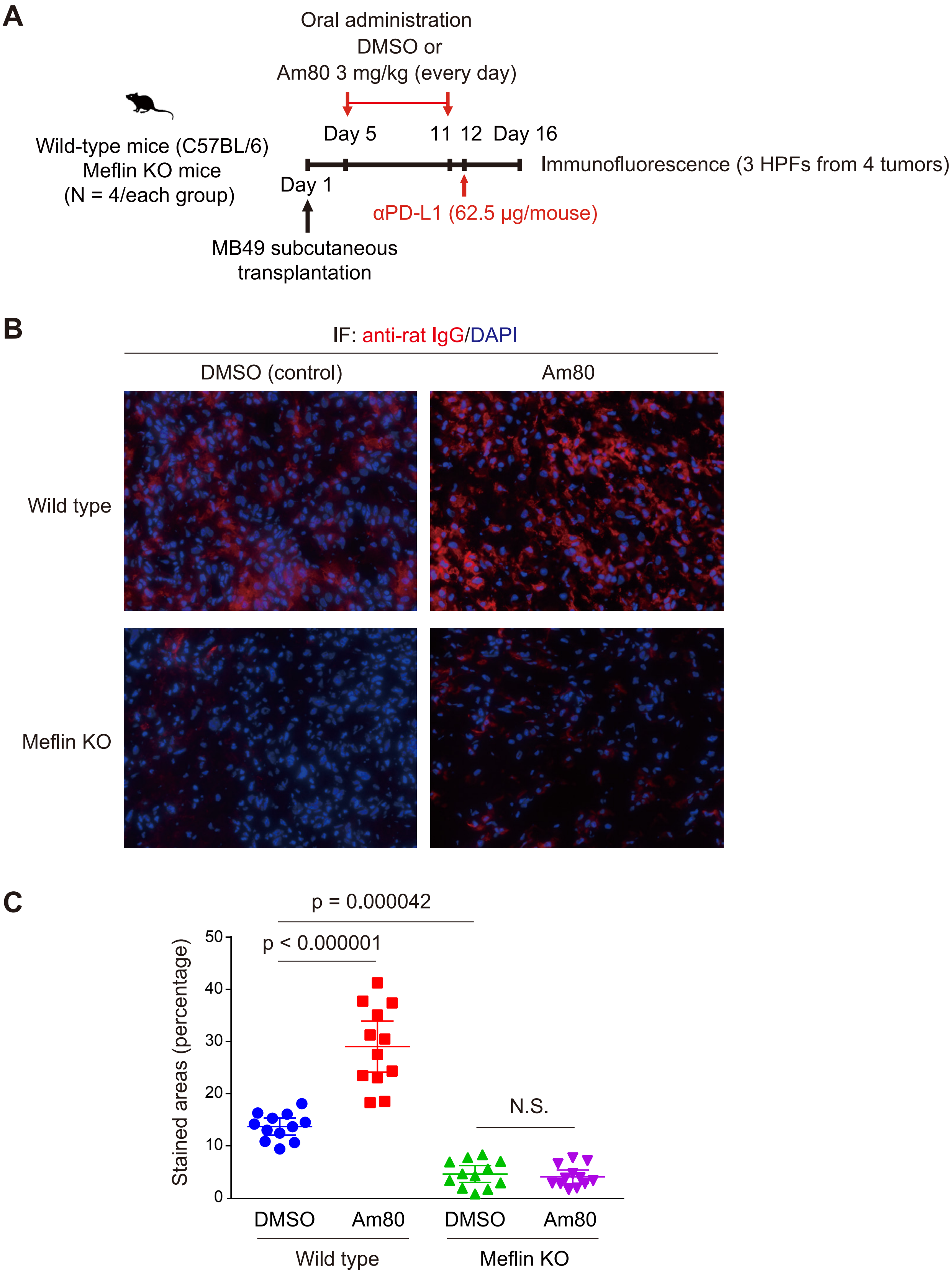

Supplementary Figure S6

Supplementary Fig. S6. Effects of oral Am80 administration on intratumoral delivery of anti-PD-L1 antibody therapy in the MB49 model.

(A) C57BL/6 wild-type or Meflin KO female mice were subcutaneously transplanted with MB49 cells (1×10^6 cells/mouse), followed by oral administration of DMSO or Am80 and subsequent intraperitoneal injection of anti-PD-L1 antibodies (rat IgG2b, 62.5 µg/mouse)

(B) Frozen sections prepared from MB49 tumors were subjected to immunofluorescent staining using anti-rat IgG antibody (red) to detect the intratumoral delivery of anti-PD-L1 antibodies. Red cells denote cells bound by anti-PD-L1 antibodies, irrespective of whether they are tumor cells or macrophages. The nuclei of all cells were visualized by DAPI staining (blue).

(C) The areas stained with anti-rat IgG antibodies were counted for the indicated groups, followed by quantification.

Statistical analysis was performed using 1-way ANOVA with Tukey test (**C**). *Ig: immunoglobulin; KO: knockout; PD-L1: programmed cell death ligand 1.*

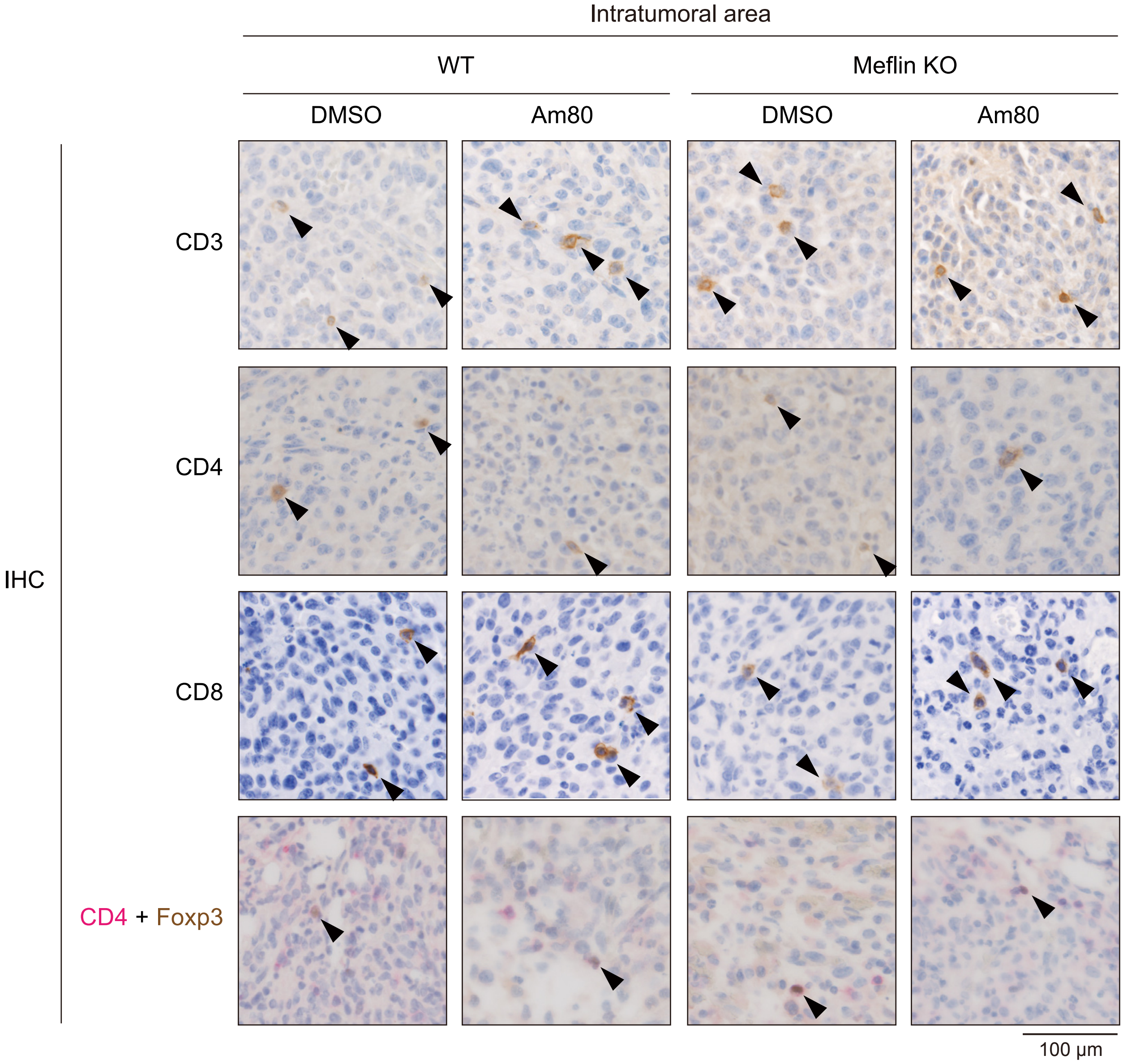

Supplementary Figure S7

Supplementary Fig. S7. Evaluation of the infiltration of T cells in the MB49 model.

Tissue sections prepared from MB49 tumors developed in wild-type (WT) or Meflin knockout (KO) mice orally administered DMSO or Am80 were stained for the indicated T cell markers. Representative images of each staining are shown. The stained positive cells found in the intratumoral, but not peritumoral, areas were selectively evaluated for quantification. For the detection of $CD4^{+}FoxP3^{+}$ T cells, a serial detection system based on the combination of diaminobenzidine (FoxP3, brown) and alkaline phosphatase (CD4, red) as chromophores was employed. Arrowheads denote positive cells.

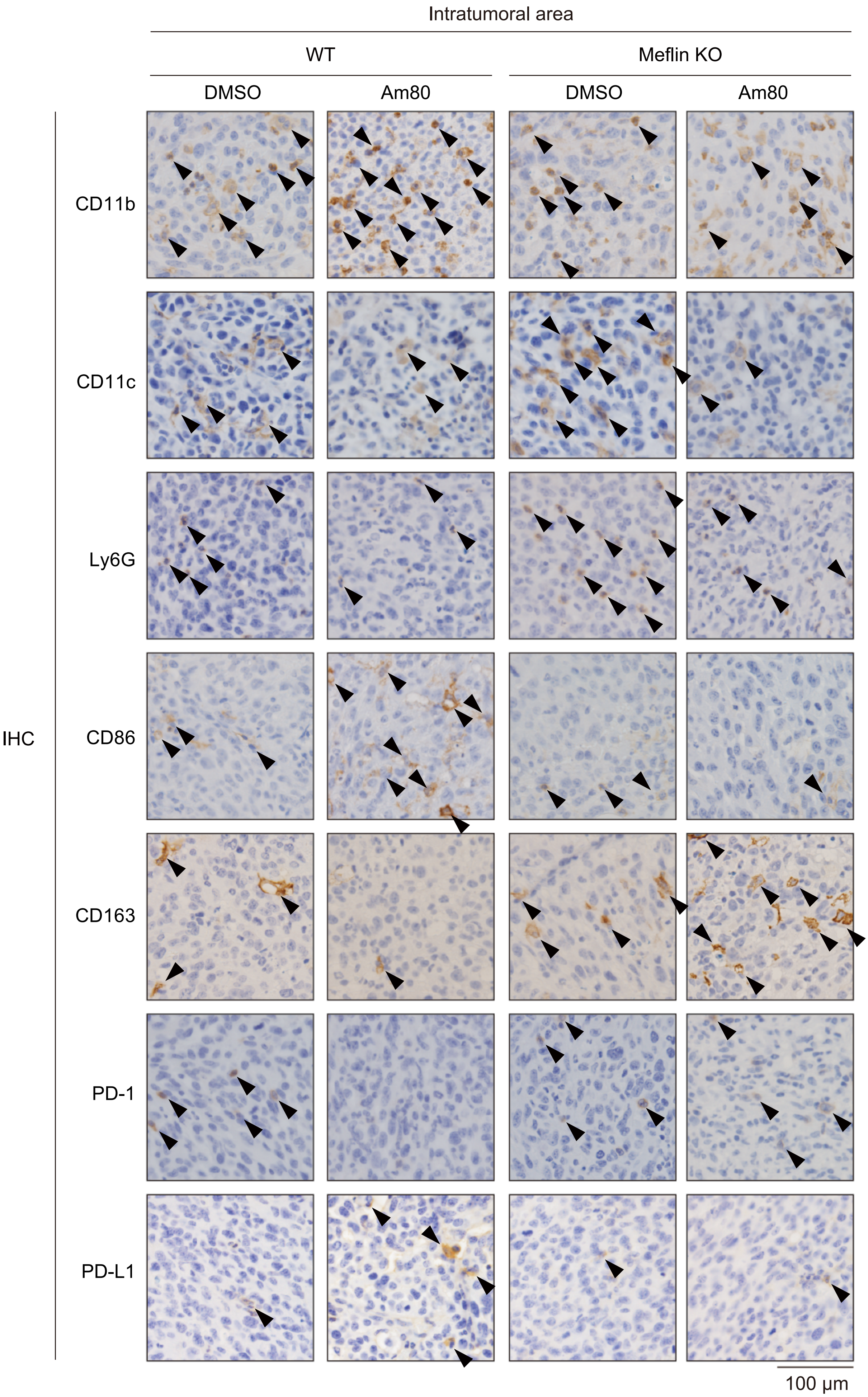

Supplementary Figure S8

Supplementary Fig. S8. Evaluation of the infiltration of myeloid cells and cells positive for PD-1 and PD-L1 in the MB49 model.

Tissue sections prepared from MB49 tumors developed in wild-type (WT) or Meflin knockout (KO) mice which received orally administration of DMSO or Am80 were stained for the indicated markers. Representative images of each staining are shown. The stained positive cells found in the intratumoral, but not peritumoral, areas were selectively evaluated for quantification. *PD-1: programmed cell death protein 1; PD-L1: programmed cell death ligand 1.*

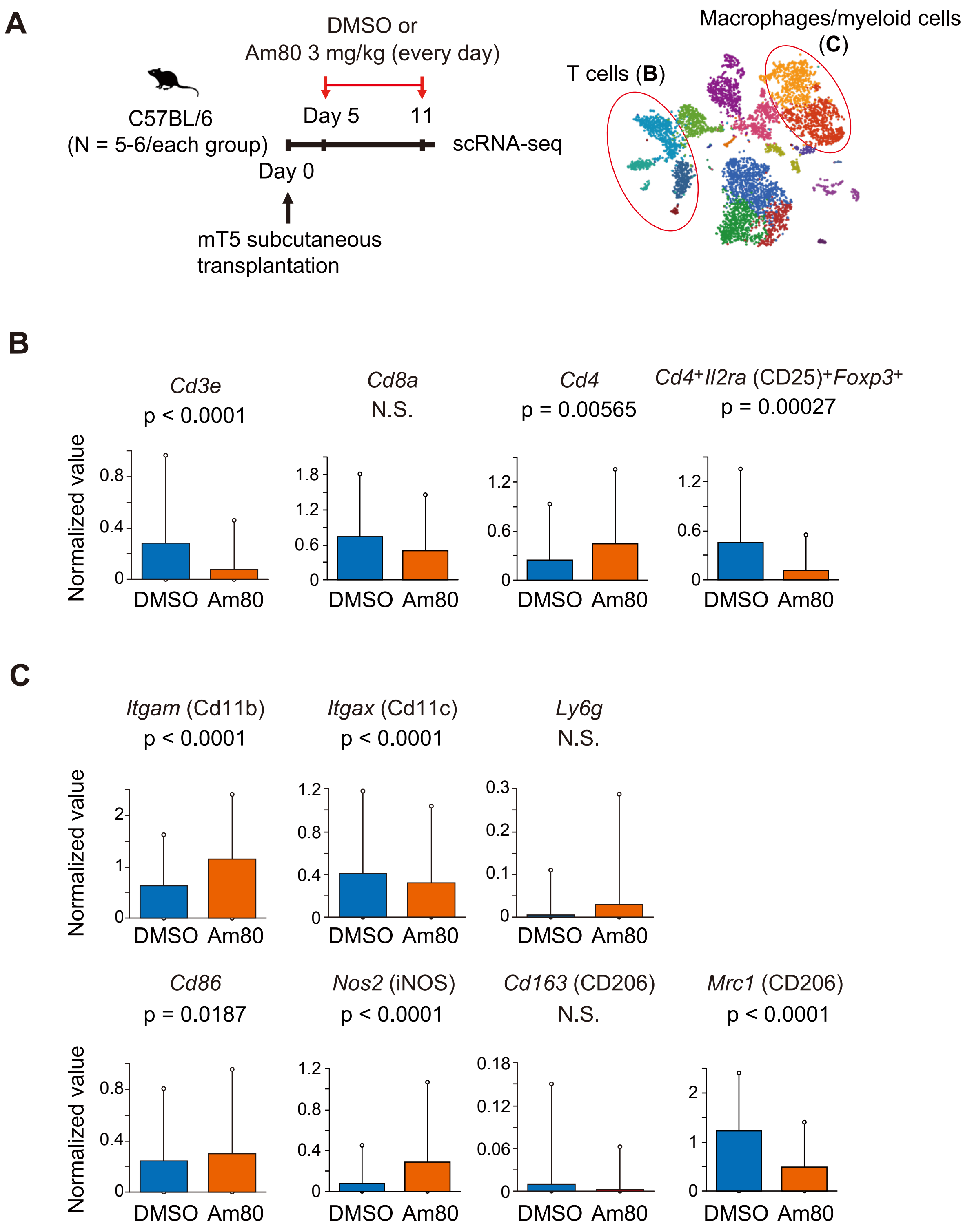

Supplementary Figure S9

Supplementary Fig. S9. Evaluation of the infiltration of immune cells in the mT5 model by single-cell transcriptomic analysis.

(A) C57BL/6 wild-type female mice were subcutaneously transplanted with mT5 cells (1×10^6 cells/mouse), followed by oral administration of DMSO or Am80 every day for the indicated period. Single-cell suspensions prepared from the developed tumors were subjected to single-cell RNA sequencing using the 10× single cell sequencing platform, followed by analysis using BBrowser software from BioTurning. Shown in the top right panel is a combined uniform manifold approximation and projection visualization of transcriptomes of all cells prepared from the DMSO and Am80 groups. T cell and macrophage clusters are indicated by circles.

(B) Expressions levels of *Cd3e*, *Cd8a*, and *Cd4* in T cell clusters of the DMSO and Am80 groups were analyzed and quantified by BBrowser. For the analysis of $Cd4^{+}Il2ra^{+}FoxP3^{+}$ cells, cells that express all three genes at the levels arbitrarily determined by BBrowser were counted and quantified.

(C) Expressions levels of the indicated genes in macrophage clusters of the DMSO and Am80 groups were analyzed by BBrowser, followed by quantification.

Statistical analysis was performed using the Welch's t-test (**B**, **C**).

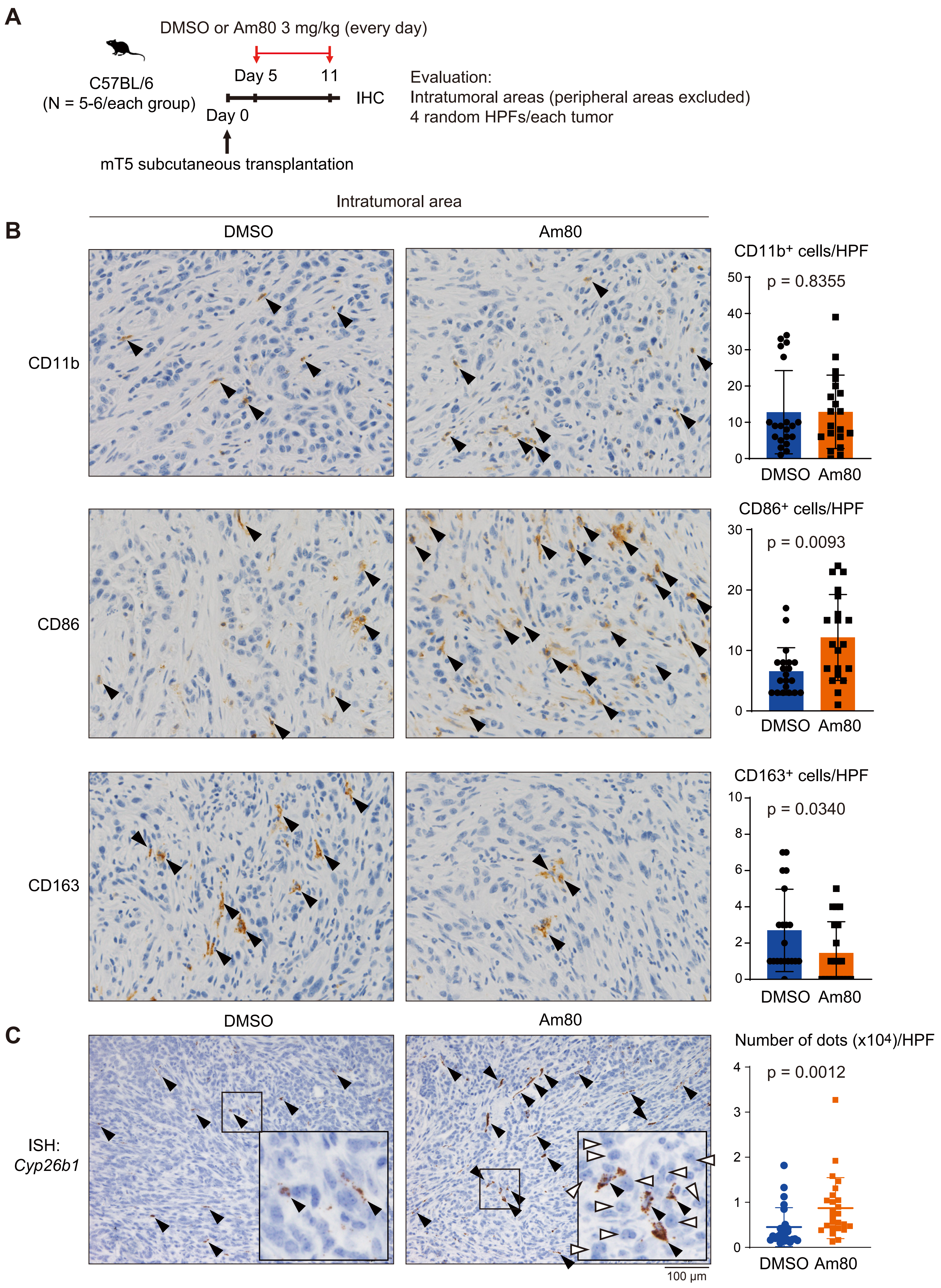

Supplementary Figure S10

Supplementary Fig. S10. Evaluation of the infiltration of myeloid cells and stromal cells with active retinoic acid signaling in the mT5 model.

(A) C57BL/6 wild-type female mice were subcutaneously transplanted with mT5 cells (1×10^6 cells/mouse), followed by oral administration of DMSO or Am80 for the indicated period, after which tissues were obtained and immunohistochemistry (IHC) was performed.

(B) Tissue sections prepared from the mT5 tumors developed in wild-type mice orally administered DMSO or Am80 were stained for the indicated myeloid cell markers by IHC **(B)** or *Cyp26b1* by ISH **(C)**. Representative images of each staining are shown (left panels). The stained positive cells found in the intratumoral, but not peritumoral, areas were evaluated, followed by quantification (right panels). In **(C)**, boxed areas are magnified in insets. Black and white arrowheads denote *Cyp26b1*$^+$ cells and tumor cells, respectively.

Statistical analysis was performed using the Welch's t-test (**B**, **C**). *IHC: immunohistochemistry; ISH: in situ hybridization.*

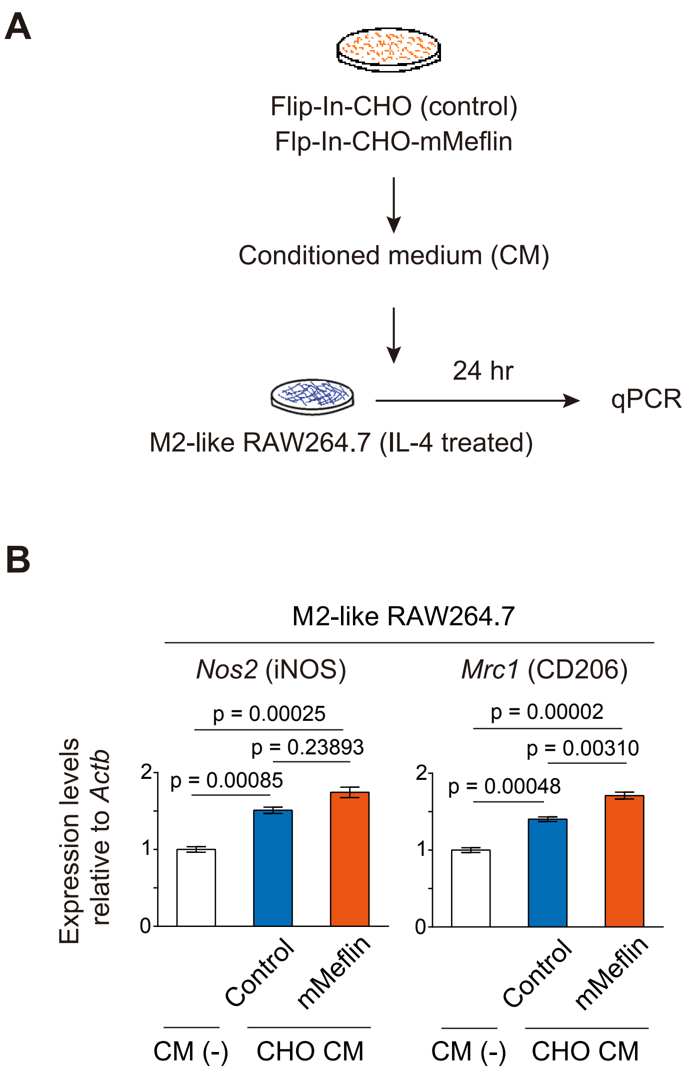

Supplementary Figure S11

Supplementary Fig. S11. No apparent effects of Meflin on the phenotype of RAW264.7 cells *in vitro*.

(A) Conditioned media (CM) of control Flp-In CHO cells or those stably expressing mouse Meflin (mMeflin) were added to RAW264.7 cells polarized to M2-like phenotype by IL-4 (40 ng/ml, 48 h). Cells were harvested and subjected to qPCR.

(B) Effects of CMs of control Flp-In CHO cells or those stably expressing mMeflin on the expression of the indicated macrophage markers in M2-like RAW264.7 cells as examined by qPCR. Each value was normalized against that of *Actb*.

Differences were assessed by 1-way ANOVA with the Tukey test (**B, C**). *qPCR: quantitative polymerase chain reaction*.

A

human PDAC, ISH: *ISLR* (Meflin)/*RARRES2*

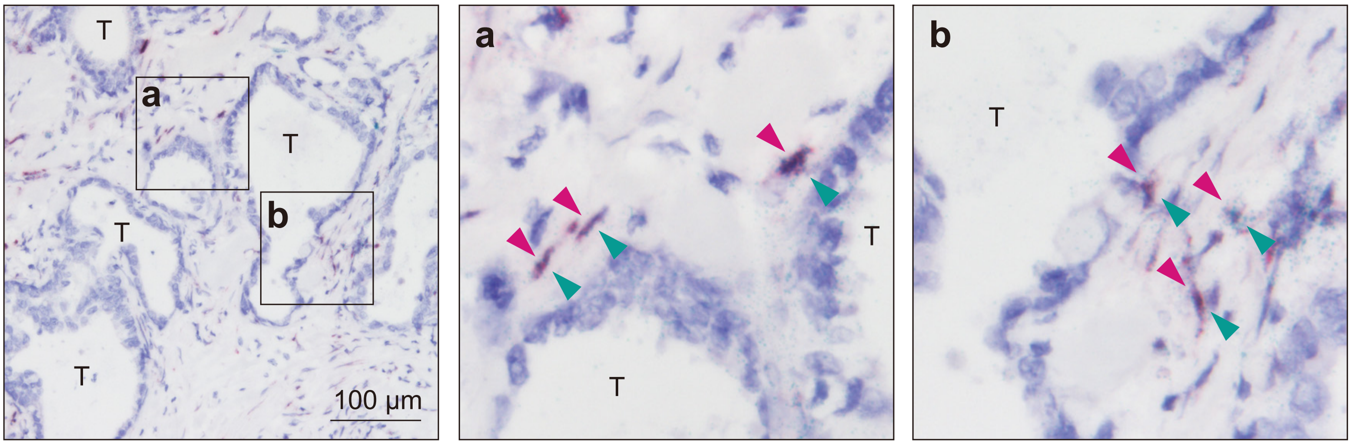

B

Cells positive for *ISLR* (Meflin) or *RARRES2*

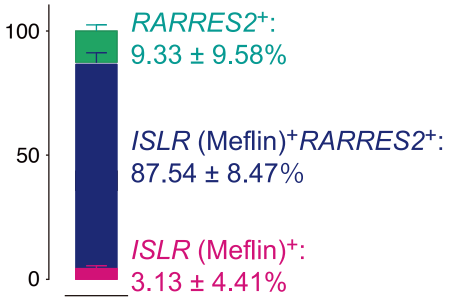

C

human PDAC, ISH: *ISLR* (Meflin)/*RARRES2*

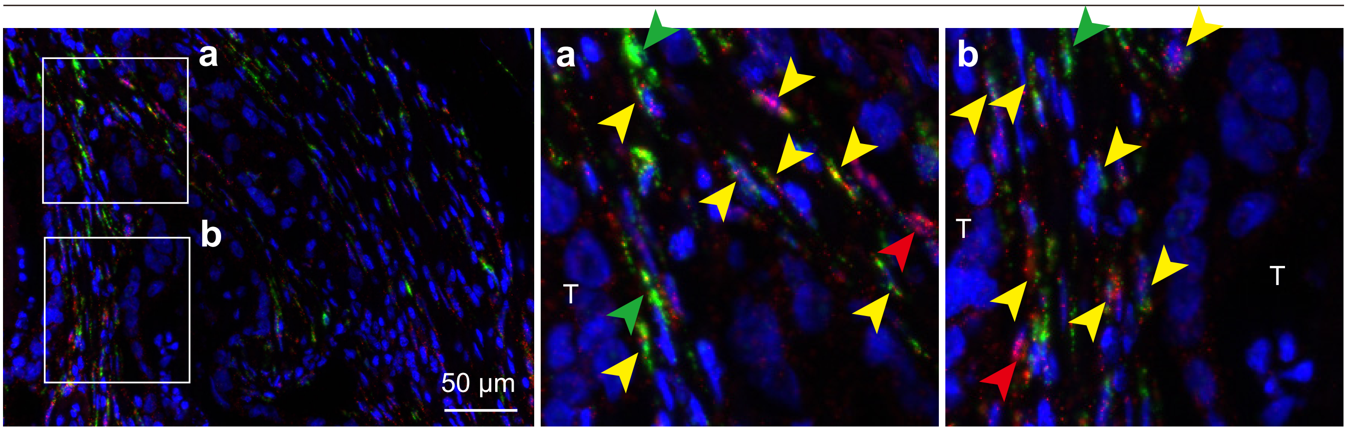

Supplementary Figure S12

Supplementary Fig. S12. *RARRES2* expression in Meflin^{+} CAFs in human PDAC.

(A, B) Co-expression of *ISLR* (Meflin) and *RARRES2* in CAFs in human PDAC. Tissue sections from patients with PDAC were double stained for *ISLR* and *Rarres2* by ISH **(A)**. Boxed areas (**a, b**) were magnified in adjacent panels. Magenta and cyan arrowheads denote *ISLR* and *RARRES2* signals, respectively. The numbers of cells single- or double-positive for *ISLR* and *RARRES2* were counted and quantified **(B)**. *T: tumor glands.*

(C) Tissue sections from patients with PDAC were subjected to duplex fluorescent ISH to double stain for *ISLR* (Meflin, green) and *RARRES2* (red). Box areas (**a, b**) were magnified in adjacent panels. Yellow arrowheads denote *ISLR* and *RARRES2* double-positive CAFs. *T: tumor glands.*

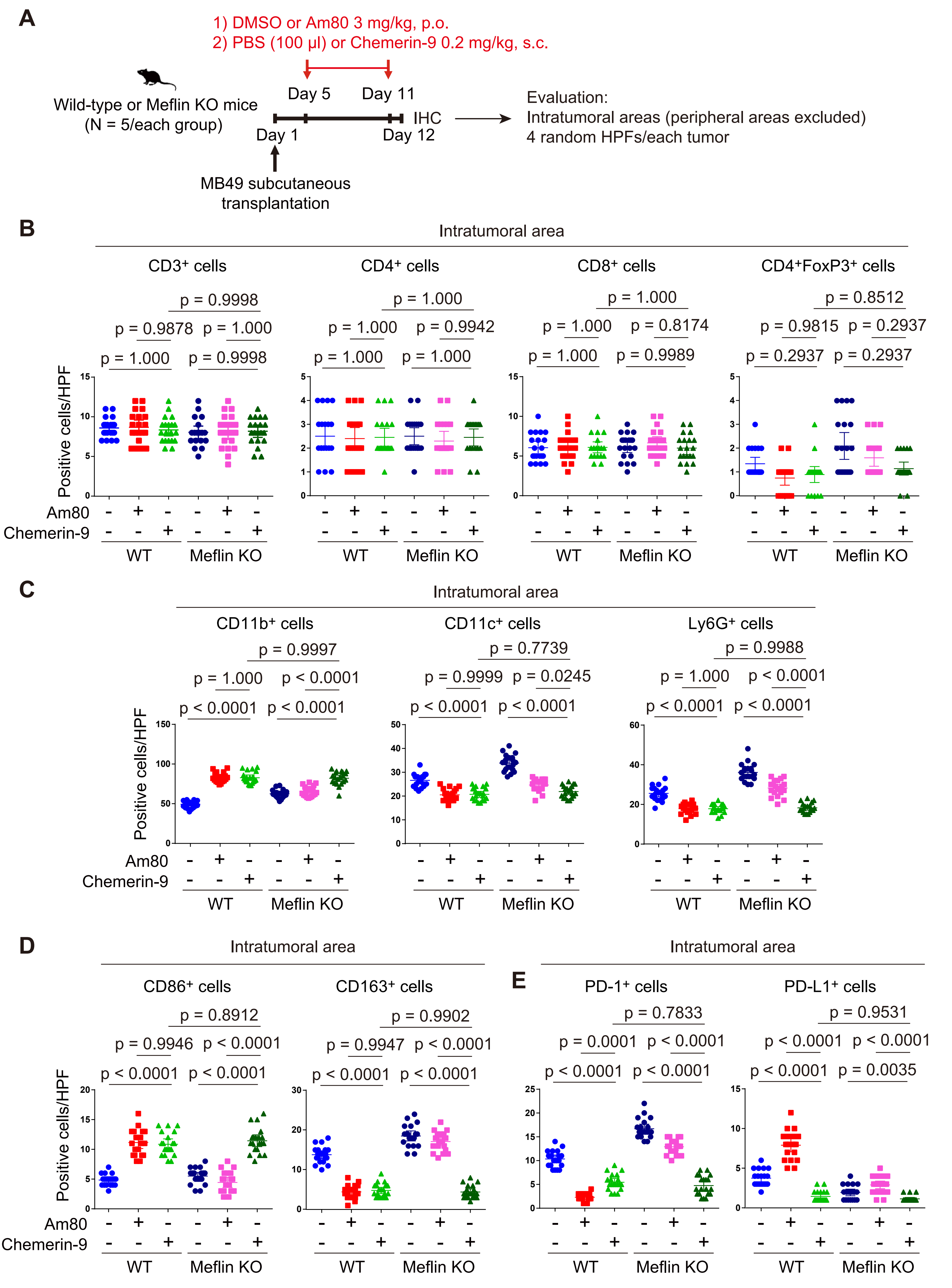

Supplementary Figure S13

Supplementary Fig. S13. Effects of chemerin-9 administration on the tumor immune microenvironment in the MB49 model.

(A) C57BL/6 wild-type or Meflin KO female mice were subcutaneously transplanted with MB49 cells (1×10^6 cells/mouse) on Day 1, followed by oral administration of DMSO or Am80 and intratumoral injection (subcutaneous injection) of PBS or chemerin-9 (0.2 mg/kg) every day during Day 5 to Day 11. The developed tumors were harvested on Day 12 and subjected to IHC to evaluate the infiltration of immune cells.

(B, C, D) Tissue sections prepared from the MB49 tumors were stained for the indicated markers by IHC. The stained positive cells found in the intratumoral, but not peritumoral, areas were evaluated, followed by quantification.

The statistical methods used are 1-way ANOVA with the Tukey test (**B-E**).

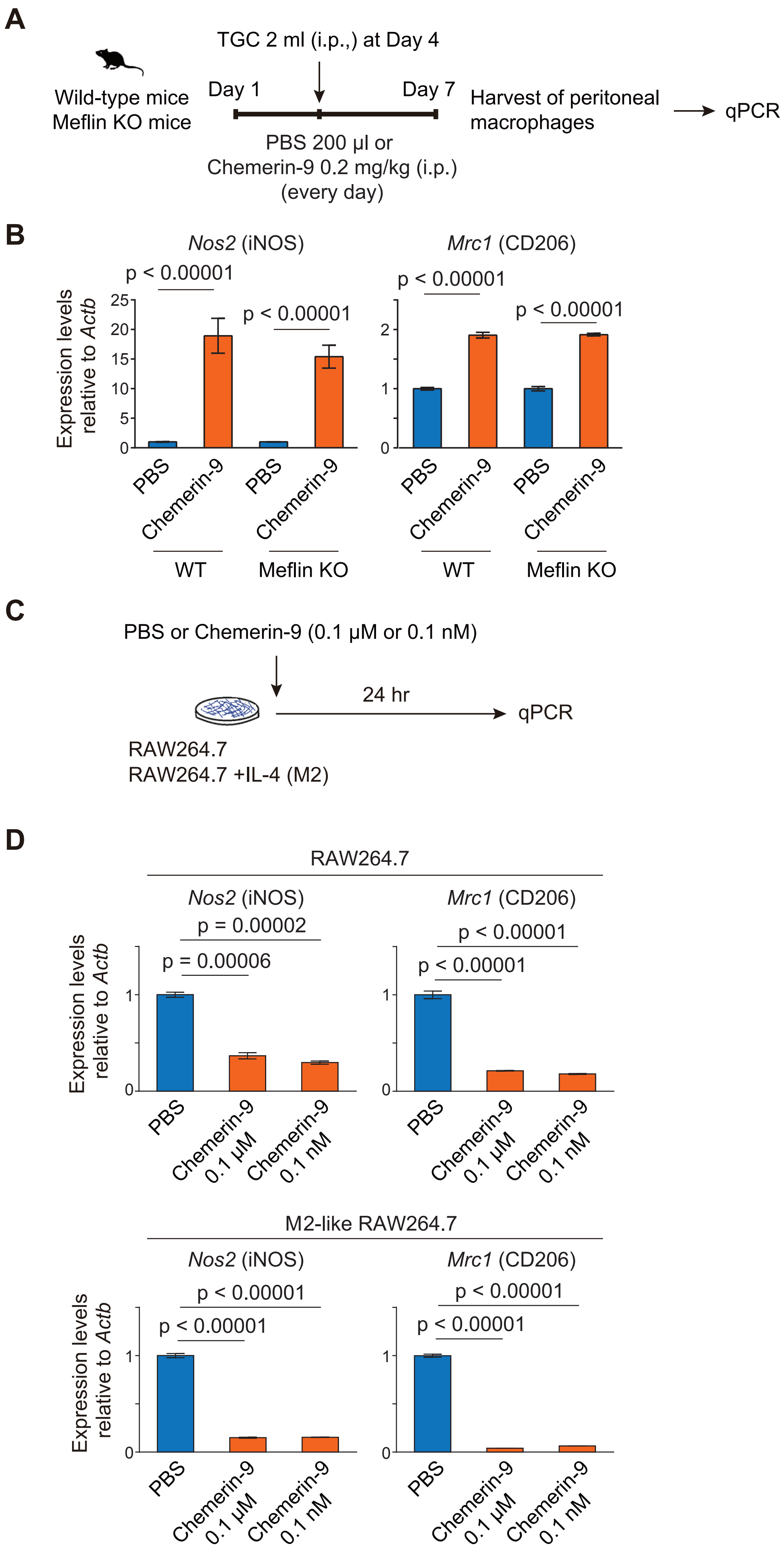

Supplementary Figure S14

Supplementary Fig. S14. Chemerin-9 induces macrophage polarization independently of Meflin expression in fibroblasts.

(A) C57BL/6 wild-type or Meflin KO female mice were intraperitoneally (i.p.) administered PBS or chemerin-9 (0.2 mg/kg) every day from Day 1 to Day 7. The mice were subjected to i.p. injection of 2 ml TGC (3%) on Day 4, followed by isolation of peritoneal macrophages on Day 7 and quantitative PCR (qPCR).

(B) Expression levels of *Nos2* and *Mrc1* relative to *Actb* in macrophages of the indicated groups were examined by qPCR.

(C) PBS or chemerin-9 (0.1 μM or 0.1 nM) were added to control RAW264.7 cells or RAW264.7 cells that had differentiated into M2-type macrophages by recombinant IL-4 (40 ng/ml, 48 h), followed by culture for 24 h and qPCR.

(D) Expression levels of *Nos2* and *Mrc1* relative to *Actb* in RAW264.7 cells of the indicated groups were examined by qPCR.

The statistical methods used are 1-way ANOVA with the Tukey test (**B**) and 1-way ANOVA with the Dunnett's test (**D**). *C-9: chemerin-9.*

Supplementary Methods

Design of animal experiments

Male mice were excluded from the present study. We did not perform a priori power calculations to determine sample size (animal numbers) for the subcutaneous tumor models, as this would require preliminary data showing a coefficient of variation and magnitude of the effect of Meflin deficiency or Am80 administration in each experimental model, which were not available in our exploratory analysis. Animals were randomly assigned to experimental groups and were operated on in random order. Animals which died within 12 h due to anesthetic problems were excluded from the studies.

In situ hybridization (ISH)

All ISH analyses were performed on formalin-fixed paraffin-embedded (FFPE) human and mouse tissue samples using RNAscope technology (RNAscope 2.5 HD Detection Kit; Advanced Cell Diagnostics) and custom-designed probes following the manufacturer's instructions. Briefly, tissue sections were baked in a dry oven (HybEZ II Hybridization System; Advanced Cell Diagnostics) at 60°C for 1 h, deparaffinized, and incubated with H_2O_2 solution (Pretreat 1 buffer) for 10 min at room temperature (RT). Slides were boiled in a target retrieval solution (Pretreat 2 buffer) for 15 min and incubated with a protease solution (Pretreat 3 buffer) for 30 min at 40°C. Slides were then incubated with the relevant probes for 2 h at 40°C and successively incubated with Amp1–6 reagents (Advanced Cell Diagnostics). Staining was visualized with 3,3-diaminobenzidine (DAB), followed by counterstaining with hematoxylin. For the detection of two genes on the same slides, we used an RNAscope 2.5 Duplex Detection Kit (Fast Green and Fast Red, Advanced Cell Diagnostics). The RNAscope probes used in this study were human Meflin (*ISLR*; NM_ 005545.3, region 275–1322, cat. no. 455481), human *RARRES2* (NM_002889.3, region 4 - 754, cat. no. 457921), mouse Meflin (*Islr*; NM_012043.4, region 763–1690, cat. no. 450041), mouse *Rarres2*

(NM_001347167.1, region 24-520, cat. no. 572581), and mouse *Cyp26b1* (NM_175475.3, region 460 - 1308, cat. no. 454241).

Assessment of ISH staining

To assess Meflin expression in the stroma, we first counted the number of cells with spindle-shaped nuclei as part of the total stromal cell count, as previously described (12). The Meflin-positive cells were then counted and divided by the total stromal cells to calculate the percentage in the stroma. When cells had four or more dots or had any clusters of ISH signals, we considered them positive. We semi-quantitatively scored the expression of Meflin in each patient according to the percentage of Meflin-positive cells. Specifically, 0% and 1–5% stromal cells expressing Meflin were combined into the score “<5” which referred to “0”, thereafter, we scored 5–10% as “5,” 10–15% as “10,” 15–20% as “15” and so forth, except the score “30<” as “30”.

Immunohistochemistry

FFPE tissue sections were deparaffinized, followed by antigen retrieval by boiling the samples in Target-Retrieval Solution (Dako) at pH 6, 7, or 9 for 30 min and conventional staining procedures, as described previously (18, 25).

Antibodies

The antibodies used in this study included rabbit monoclonal anti-CD3 antibodies (clone SP7, cat. no. ab16669, dilution 1:100; Abcam), rat monoclonal anti-CD4 antibodies (clone 4SM95, cat. no. 14-9766-82, dilution 1:100; eBioscience), rabbit monoclonal anti-CD8α antibodies (clone D4W2Z, cat. no. 98941, dilution 1:800; Cell Signaling Technology), rabbit monoclonal anti-CD11b antibodies (clone EP45, cat. no. AC-0043RUO, dilution 1:200;

Epitomics), rabbit monoclonal anti-CD11c antibodies (clone D1V9Y, cat. no. 97585, dilution 1:200; Cell Signaling Technology), rabbit monoclonal anti-Ly6G antibodies (clone E6Z1T, cat. no. 87048, dilution 1:200; Cell Signaling Technology), rabbit monoclonal anti-CD86 antibodies (clone E5W6H, cat. no. 19589, dilution 1:200; Cell Signaling Technology), rabbit monoclonal anti-FoxP3 antibodies (clone D6O8R, cat. no. 12653, dilution 1:200; Cell Signaling Technology), rabbit monoclonal anti-PD-1 antibodies (clone D7D5W, cat. no. 84651, dilution 1:400; Cell Signaling Technology), and rabbit monoclonal anti-PD-L1 antibodies (clone D5V3B, cat. no. 64988, dilution 1:200; Cell Signaling Technology), mouse monoclonal anti-CD68 antibodies (clone PGM-1, cat. no. M0876, dilution 1:100; Dako) and rabbit monoclonal anti-CD163 antibodies (clone EPR19518, cat. no. ab182422, dilution 1:500; Abcam).

Fluorescent immunostaining

Fluorescent immunostaining was performed on frozen sections prepared from mouse tumor tissue samples. After incubation with 10% neutral buffered formaldehyde solution (cat. no. 37152-51; Nakalai) for 5 min at RT, the slides were washed twice. For staining with anti-mPD-L1, the slides were incubated with Alexa 594-conjugated donkey anti-rat IgG (cat. no. A21209; Thermo Fisher Scientific) for 1 h at RT. Nuclei were visualized using DAPI staining. Images were acquired and quantified with a fluorescent microscope equipped with a CCD camera (BZ-X710; KEYENCE).

Quantitative polymerase chain reaction (qPCR)

Total RNA was purified from TGC-induced peritoneal macrophages and cultured cells using the RNeasy Mini Kit (Cat. no. 74104; QIAGEN) according to the manufacturer's instructions. Purified RNA samples were reverse-transcribed using ReverTra Ace (Cat. no. TRT-101; Toyobo) with oligo dT and random primers. qPCR of the generated cDNA was

performed with TaqMan Gene Expression Master Mix (Cat. no. 4369016; Applied Biosystems) on an Mx3005P thermal cycler (Agilent Technologies). TaqMan probes and primers for mouse *Nos2* (Mm00440502_m1), mouse *Mrc1* (Mm01329359_m1), and mouse β-actin (*Actb*; Mm02619580_g1) were purchased from Thermo Fisher Scientific and used according to the manufacturer's instructions. Cycling conditions were as follows: 95 °C for 10 min, 40 cycles of 95 °C for 15 s, and then 60 °C for 1 min, followed by one cycle of 95 °C for 10 s. The data were analyzed using the $2^{-\Delta\Delta ct}$ method and normalized to *Actb* control.

Sample preparation for single-cell RNA sequence

Tumor specimens were minced and enzymatically digested in DMEM supplemented with bovine serum albumin (BSA, 2 mg/mL, Sigma, A7906-100G), Liberase TL (150 μg/mL, Sigma #540120001), and DNase I (25 μg/mL, Roche, #11284932001) for 60 min at 37°C with agitation. Cell digestion was strained through a 100-μm cell strainer and resuspended in PBS containing 0.04% BSA. Cells were counted on Countess automated cell counter (Thermo Fisher), and 12,000 viable cells were loaded per lane on 10X Chromium microfluidic chips (10X Genomics). Single-cell capture, barcoding, and library preparation were performed using the 10X Chromium version 2 chemistry, according to the manufacturer's protocol (10X Genomics, #CG00052). cDNA libraries were checked for quality on Agilent 4200 Tapestation and quantified by KAPA qPCR, followed by sequencing by GENEWIZ on a single lane of a HiSeq4000 (Illumina) to an average depth of 50,000 reads per cell.

Single-cell data processing and analysis

Publicly available single-cell RNA sequencing data from human bladder UC (GSA acc.no HRA000212, ref. 30) and human PDAC (GSA acc. No. CRA001160, ref. 40) were analyzed and visualized using the Bioturing BBrowser. For the analysis of single-cell RNA sequencing

data obtained from mT5 tumors, we utilized the Cell Ranger pipeline (v1.3, 10X Genomics) for processing Illumina sequencing data, which involved converting base call files to FASTQ format, aligning FASTQ files to mouse (mm10) reference genomes, and generating digital gene-cell counts matrices. The samples were integrated using the Cell Ranger aggregate function to standardize the number of transcriptomic reads across samples. After these processes, expression analysis of macrophages and T cells was conducted using the BBrowser.
